# Alterations in SLC4A2, SLC26A7 and SLC26A9 Drive Acid–Base Imbalance in Gastric Neuroendocrine Tumors and Uncover a Novel Mechanism for a Co-Occurring Polyautoimmune Scenario

**DOI:** 10.3390/cells10123500

**Published:** 2021-12-10

**Authors:** Oriol Calvete, José Reyes, Hernán Valdés-Socin, Paloma Martin, Mónica Marazuela, Alicia Barroso, Javier Escalada, Antoni Castells, Raúl Torres-Ruiz, Sandra Rodríguez-Perales, María Currás-Freixes, Javier Benítez

**Affiliations:** 1 Human Genetics Group, Spanish National Cancer Research Center (CNIO), 28029 Madrid, Spain; p.martingimeno@hotmail.com (P.M.); abarroso@cnio.es (A.B.); 2 Network of Research on Rare Diseases (CIBERER), 28029 Madrid, Spain; 3 Grupo Español de Tumores Neuroendocrinos y Endocrinos (GETNE), 28054 Madrid, Spain; jose.reyes@hcin.es; 4 Department of Gastroenterology, Hospital Comarcal de Inca, 07300 Inca, Spain; 5 Health Investigation Institute (IDISBA), 07120 Palma de Mallorca, Spain; 6 Department of Endocrinology, Centre Hospitalier Universitaire de Liège, 4000 Liège, Belgium; hg.valdessocin@chuliege.be; 7 Hospital la Princesa, Instituto de Investigación Princesa, University Autónoma of Madrid, 28006 Madrid, Spain; monica.marazuela@gmail.com; 8 Endocrinology and Nutrition Department, Clínica Universidad de Navarra, 31008 Pamplona, Spain; fescalada@unav.es; 9 IdiSNA, Navarra Institute for Health Research, 31008 Pamplona, Spain; 10 CIBER Fisiopatología de la Obesidad y Nutrición (CIBEROBN), Instituto de Salud Carlos III, 28009 Madrid, Spain; 11 Hospital Clinic of Barcelona, IDIBAPS, CIBEREHD, University of Barcelona, 08036 Barcelona, Spain; castells@clinic.cat; 12 Molecular Cytogenetics and Genome Editing Unit, Spanish National Cancer Research Center (CNIO), 28029 Madrid, Spain; rtorresr@cnio.es (R.T.-R.); srodriguezp@cnio.es (S.R.-P.); 13 Endocrinology and Nutrition Department, Clínica Universidad de Navarra, 28027 Madrid, Spain; mcurras@unav.es

**Keywords:** gastric neuroendocrine tumors (gNETS), autoimmune thyrogastric syndrome, autoimmune polyendocrine syndrome (APS), achlorhydria, solute carriers (SLCs), immunodeficiencies

## Abstract

Autoimmune polyendocrine syndrome (APS) is assumed to involve an immune system malfunction and entails several autoimmune diseases co-occurring in different tissues of the same patient; however, they are orphans of its accurate diagnosis, as its genetic basis and pathogenic mechanism are not understood. Our previous studies uncovered alterations in the ATPase H+/K+ Transporting Subunit Alpha (ATP4A) proton pump that triggered an internal cell acid–base imbalance, offering an autoimmune scenario for atrophic gastritis and gastric neuroendocrine tumors with secondary autoimmune pathologies. Here, we propose the genetic exploration of APS involving gastric disease to understand the underlying pathogenic mechanism of the polyautoimmune scenario. The whole exome sequencing (WES) study of five autoimmune thyrogastric families uncovered different pathogenic variants in SLC4A2, SLC26A7 and SLC26A9, which cotransport together with ATP4A. Exploratory in vitro studies suggested that the uncovered genes were involved in a pathogenic mechanism based on the alteration of the acid–base balance. Thus, we built a custom gene panel with 12 genes based on the suggested mechanism to evaluate a new series of 69 APS patients. In total, 64 filtered putatively damaging variants in the 12 genes of the panel were found in 54.17% of the studied patients and none of the healthy controls. Our studies reveal a constellation of solute carriers that co-express in the tissues affected with different autoimmune diseases, proposing a unique genetic origin for co-occurring pathologies. These results settle a new-fangled genetics-based mechanism for polyautoimmunity that explains not only gastric disease, but also thyrogastric pathology and disease co-occurrence in APS that are different from clinical incidental findings. This opens a new window leading to the prediction and diagnosis of co-occurring autoimmune diseases and clinical management of patients.

## 1. Introduction

**Figure 1:**
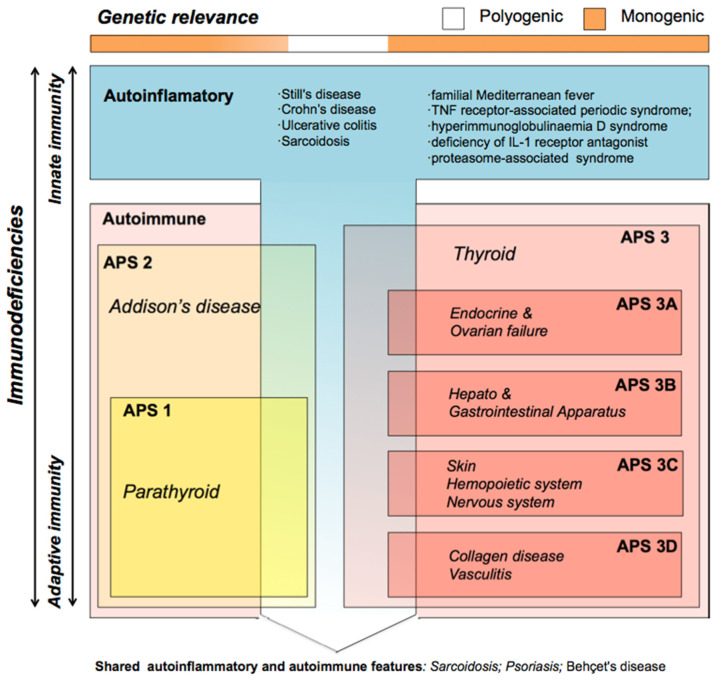
Classification of immunodeficiencies adapted from Neufeld and Blizzard (1980) based on clinical criteria only. The genetic origin of autoinflammatory and autoimmune alterations is considered monogenic (orange in the genetic relevance bar) when they are well compartmentalized, but compatible with polygenic models (white in the genetic relevance bar) when the immune disease shares components from innate and adaptive immunity. Different autoimmune diseases often co-occur in the same patient (autoimmune polyendocrine syndrome, APS); there are different APS types, APS1 and 2 involve Addison’s disease (yellow box), while APS3 (red box) involves thyroid disease (both hypothyroidism and hyperthyroidism). APS3 patients are subclassified depending on the tissues of origin of the co-occurring APS pathologies; they typically have co-occurring endocrine and ovarian (APS3A); hepatic and gastrointestinal (APS3B); skin, hematopoietic and nervous system (APS3C); or collagen and vasculitis (APS3D) autoimmune diseases.

Immunodeficiencies are alterations of the immune system that can lead to autoinflammatory or autoimmune processes that, when persisting over time, can progress towards cell atrophy and neoplasia. Autoinflammatory diseases are considered to be dependent on the malfunction of the rapid innate immune system [1], while autoimmune diseases arise from the malfunction of the adaptive immune system [2]. To date, over 60 human diseases are known that belong to this latter group, but specific treatments and diagnostic guidelines are still lacking for most of them [3]. Different autoimmune diseases are often found co-occurring in the same patient or in family members (autoimmune polyendocrine syndrome, APS), and a marked but unknown genetic component appears to be involved [4]. Current APS subclassification is based on clinical criteria and frequencies of co-occurrence, but genetic criteria are not considered (Figure 1) [5]. APS type 3, in which thyroiditis represents the pivotal disorder, typically co-occurs with endocrine (subtype 3A), gastrointestinal (subtype 3B), skin and nervous system (subtype 3C), or collagen (subtype 3D) autoimmune diseases. When these pathologies are well compartmentalized within innate or adaptive response malfunction, they are considered to be compatible with a monogenic model. Deregulation of cytokine signaling, the inflammasome or the immune response have been proposed to participate in the pathology development, although in most cases the causes and possible genes involved are not known [6]. Mutations in the autoimmune regulator (AIRE) gene [7] and different HLA alleles [3] have been associated with disorders in APS1 and APS2 patients. Mutations in the antigen 4 gene in CTLA-4, which is an important negative regulator of the activation of T cells, are involved in type 1 diabetes mellitus (DM) and autoimmune thyroid diseases (Graves’ disease and Hashimoto’s thyroiditis) [8]. Otherwise, when the etiology of the APS shares components with inflammatory response-mediated disease, it has been suggested that a polygenic model is involved [9].

In the stomach (APS3B subtype), autoimmune anemia was described as a consequence of chronic atrophic gastritis, which can progress to gastric neuroendocrine tumors (gNETs) [10]. Type I gastric NETs usually arise in patients who have autoimmune atrophic gastritis leading to an atrophy of parietal cells that results in gastric hypochlorhydria; while type II gastric NETs are not associated with achlorhydria and type III gastric NETs, arise sporadically, and are not associated with hypergastrinemia. We previously described that gastric achlorhydria was mediated by a mutation in the ATPase H+/K+ transporting alpha subunit (ATP4A) gene (patient F1, see Table 1), which encodes the proton pump expressed in parietal cells (PCs) responsible for gastric acidification, and correlates with chronic gastritis that leads to gNETs [11,12]. Our knock-in (KI) mouse model for this ATP4A mutation not only confirmed the relation of the candidate gene with achlorhydria and the progression to gNETs [13], but also suggested that the ATP4A malfunction was altering the acid–base balance within PCs, which triggered the immune response and atrophy [14]. Recently, co-occurring colitis compatible with inflammatory bowel disease was observed in the follow-up of these ATP4A malfunction-mediated gNET patients [15]), which suggested a common genetic origin of two immunodeficiencies of different etiology. Similarly, a second studied family (patient F2, see Table 1) was explained with a digenic model involving the ATP4A and the parathyroid hormone 1 receptor gene (PTH1R), which is also involved in PC differentiation. The digenic model explained not only gNETs but also the hypothyroidism and rheumatoid arthritis observed in this patient [12]. Our findings suggested a common mechanism for autoimmune gastric and thyroid disease, which are frequently found together [16].

In order to explore the common genetic origin of autoimmune thyrogastric syndrome under a polyautoimmune scenario, we have studied a new series of 74 patients affected with both gastric and thyroid diseases that were co-occurring with other immunopathies to further explore the genetic landscape of the APS. In this work, we describe a unique genetic landscape involving a novel pathogenic mechanism, which explains not only the gastric disease that leads to gNETs, but also the autoimmune thyrogastric pathology and the disease co-occurrence in APS.

## 2. Materials and Methods

### 2.1. Studied Series

A series of 76 patients was evaluated in this study. Five autoimmune thyrogastric syndrome patients (involving autoimmune gastritis or gNETs and Graves’ or Hashimoto’s disease) were included in the first gene discovery series and initially studied by Whole Exome Sequencing (Discovery WES1). Preliminary candidate genes found in this study were included in a custom achlorhydria panel for targeted Next Generation sequencing (tNGS) studies. A second series of 69 APS patients was studied with the custom panel. The efficacy of the panel was evaluated for all 74 recruited patients plus the 2 previously studied probands by WES from families F1 and F2 [11]. The Research Ethics Committee of the Hospital Universitario de Fuenlabrada (Ref: 16–62) approved this study. Written informed consent was obtained from all participants.

### 2.2. Patients

The series included 57 familial (F1 to F57) and 19 nonfamilial (S1 to S19) patients. Patients were considered familial or sporadic when the same or other immunodeficiencies were found or not found in family members, respectively. Regarding gastric diseases, 24 and 46 patients were affected with gNETs and chronic atrophic gastritis (CAG), respectively (see Table 1 for clinical details). When possible, peripheral blood samples from affected and healthy family members of the probands were also collected for further segregation studies of the candidate variants. Individuals from 18 families were recruited. Candidate variants in affected patients that were also found in healthy or asymptomatic family members were discarded for filtering strength. Forty healthy Spanish individuals with healthy first-degree relatives were collected for control purposes in the tNGS studies.

### 2.3. Quantitative RT-PCR

Five hundred nanograms of total RNA were reverse transcribed using the High-Capacity cDNA Reverse Transcription Kit (Applied Biosystems, Foster City, CA, USA) in a final volume of 20 μL using a standard protocol. Primers were designed with Primer3software (v. 0.4.0). The sequences of the primers are listed in Table S1. Quantitative RT-PCR (qRT-PCR) using the GoTaq ^®^ qPCR Master Mix (Promega, Madison, WI, USA) was performed to evaluate gene expression. Samples were amplified using the standard cycling conditions. The analysis was performed using QuantStudio™ Real-Time PCR Software v1.3.

### 2.4. Statistics

Values within the compared groups were evaluated with the Kolmogorov–Smirnov test to determine normal distribution. Student’s *t*-test was used for the comparison of normally distributed values. Differences were considered to be significant when the exact *p*-value was <0.05.

### 2.5. Discovery Series WES1

Exomes from 5 thyrogastric patients were sequenced. The detailed procedure and pipeline are described in Supplementary Materials. Sequencing data have been deposited in the RD-Connect GPAP platform from the CNAG (National Center for Genomic Analysis), available at https://platform.rd-connect.eu/genomics/ (accessed on 15 November 2021, project number 634935 included in the 2017 BBMRI-LPC Whole Exome Sequencing Call).

### 2.6. In Vitro Studies

DNA constructs and the cloning of guide RNAs for SLC26A7, SLC26A9 and SLC4A2 genes as well as lentivirus generation, titration, transduction and antibiotic selection are described in Supplementary Materials. The sgRNAs and oligo sequences are listed in Table S1. Wild type (WT) HEK293 and knock-out (KO) cell lines were cultured in an enriched medium (DMEM with 10% FBS, 1% penicillin/streptomycin and 0.5% fungizone), and a restrictive medium (the same, but without FBS), at 37 °C in a humidified 5% CO_2_ atmosphere. The colony-forming assay and flow cytometry studies are described in Supplementary Materials.

### 2.7. Targeted Next Generation Sequencing (tNGS)

We designed a custom gene panel of 12 genes for tNGS studies. The panel included our previously described genes in F1 and F2 (ATP4A and PTH1R) [11,12], and the genes uncovered in the Discovery WES1 study (SLC4A2, SLC26A7, SLC26A9 and PTH2R). Genes involved in the acid–base balance and gradient homeostasis in PCs (SLC9A2, SLC9A4, KCNE2, KCNQ1 and KCNJ16) and the gastrin receptor for the positive regulation of gastric acidification (CCKBR2) were also included in the panel. The detailed pipeline is described in Supplementary Materials.

## 3. Results

### 3.1. Thyrogastric Syndrome

Five families (F3 to F7) affected with hypothyroidism and gastric autoimmune diseases (Table 1) were recruited for WES (Discovery WES1) to further investigate the genetic landscape of autoimmune thyrogastric syndrome. An average of 2192 filtered variants were obtained per sequenced patient. All filtered variants (10,962) were included in an Induced Network Module study (CPDB) enriched with the previously described genes in patients F1 and F2 (ATP4A and PTH1R) [11,12]. Five genes entailing eight variants were found in the five families involved with ATP4A and PTH1R genes (Table S2). Concretely, the Discovery WES 1 study uncovered a new mutation in the ATP4A gene (ATP4Ap.Pro240His) and a mutation in another gene involved in the regulation of Ca^2+^ gastric absorption (PTH2Rp.Ile194Met). Putatively damaging variants in a second group of genes expressed in parietal cells (PCs), and playing an important role in achieving gastric chlorhydria along with the ATP4A proton pump, were also found in the studied families (1xSLC4A2, 2xSLC26A7 and 3xSLC26A9) (Table S2). These solute carriers are responsible for the regulation of the acid–base balance in PCs and directly contribute to H+/K+ exchange. KO mouse models for SLC26A7, SLC26A9 and SLC4A2 were described to have gastric achlorhydria [17,18,19]. In addition, hepatobiliary and immunologic changes were observed in SLC4A2 deficient mice, while no APS exploration was performed for the other two cited models. Segregation studies were completed for selected familial patients (Figure S1).

**Figure 2:**
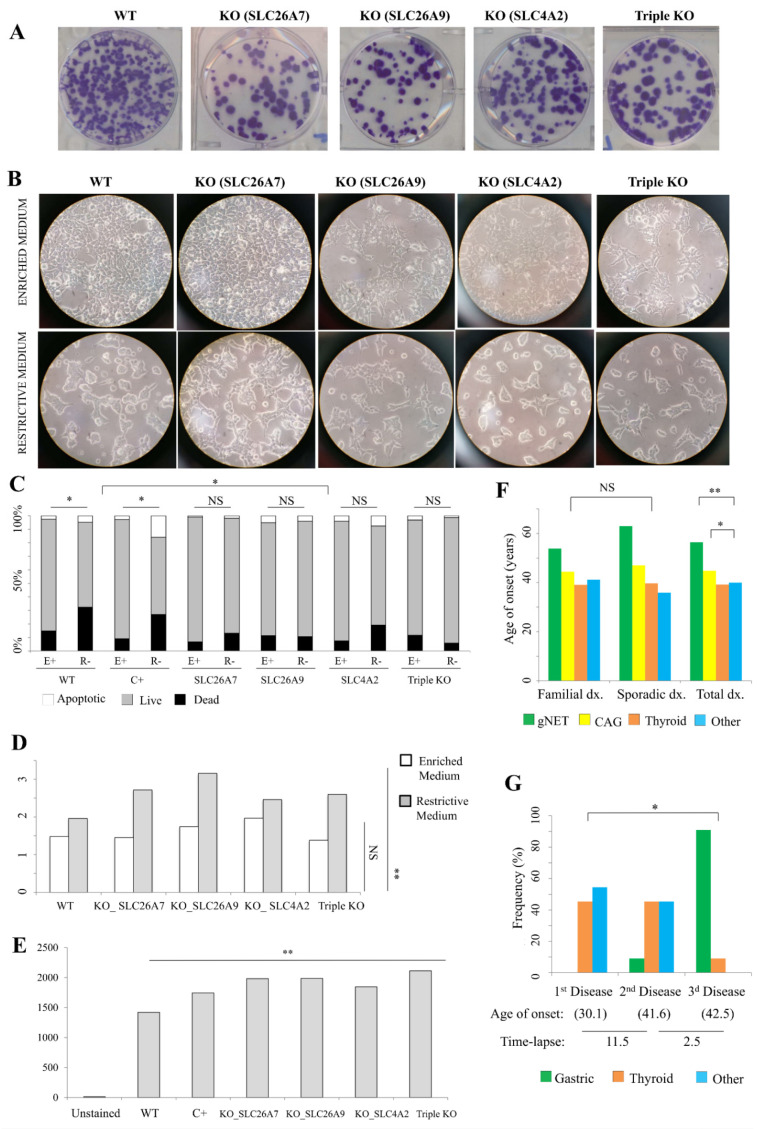
Thyrogastric gene functional studies. (**A**) Colony-forming assay. 100× wild type (WT) HEK293T cells and knock-out (KO) cells were initially seeded and stained with crystal violet at 10 days after plating. (**B**) Microscopy observation of WT HEK293T cells and KO cells grown in enriched or restrictive medium. (**C**) Flow cytometry results for WT HEK293T cells and KO cells stained with TMRE fluorescence for viability testing. H_2_O_2_ treated-cells were included as a positive control (C+) for induced-apoptosis. Cells grown in enriched (E+) and restrictive (R-) media were included in the study. Significant differences were observed in both WT and C+ cells when compared to E+ and R- medium cultures, but no differences were found between medium cultures in KO cell lines. Significantly different viability percentages were observed between WT and C+ cells compared to KO cell lines. NS: not significant; * *p* < 0.05. (**D**) Percentage (%) of SubG1 phase (apoptotic) for WT HEK293T cells and KO cells. Cells grown in enriched (E+) and restrictive (R-) media were included in the study. Significant differences were observed in the number of apoptotic cells between WT and KO cells in R- medium. NS: not significant; ** *p* < 0.01. (**E**) Flow cytometry results for WT HEK293T cells and KO cells stained with DCFDA fluorescence for ROS damage testing. H_2_O_2_ treated-cells were included as a positive control (C+) for induced-apoptosis. Significant differences were observed in the number of ROS damage-mediated apoptotic cells between WT and KO cells in R- medium. ** *p* < 0.01. (**F**) Average age of onset (dx.) of the immunodeficiencies in familial and sporadic patients (gNET or CAG, thyroid and other immunodeficiencies). No differences were found between age of onset of thyroid disease and other immunodeficiencies in familial or sporadic patients. Gastric disease (gNET and CAG) had a significantly later age of onset. * *p* < 0.05; ** *p* < 0.01. (**G**) Average age of onset for the first, second and third disease and the frequency of disease type for patients with at least 3 pathologies (N = 26). Average time-lapse between ages of onset of different immunodeficiencies is also shown. * *p* < 0.05.

On the other hand, acumulated ROS damage and increased apoptosis deregulation was observed in the PCs of mutated ATP4A patients [14]. An evaluation of the previously described acid–base imbalance for ATP4A mutations was tested in vitro for the new candidate genes. Gene expression of the candidate genes was verified in different cell lines by RT-PCR (Figure S2A). The HEK293T cell line expressed the three genes, although renal acidosis has not been reported in any KO mouse model for the selected genes [17,18,19], suggesting a compensatory mechanism. Thus, SLC26A7, SLC26A9 and SLC4A2 genes were inactivated in HEK293T cell lines by using CRISPR KO technology to evaluate the relevance of the putative defects in solute transport across the cytoplasmatic membrane in our series of thyrogastric patients. First, a colony-forming assay was performed. Significant differences were observed in the number or size of colonies in KO cell lines compared to WT cells (Figure 2A), which suggests that the lack of solute transport alters the proliferation/aggregation of these KO cell models. The morphology of the cells was evaluated in two different growth medium conditions. Less aggregated cells, and altered cytoplasm and cell morphology were found in KO cells compared to WT cells (Figure 2B) when grown in optimal enriched media, which contains a high quantity of hormones, macromolecular proteins and a variety of small molecules (amino acids, sugars and lipids). Accentuated disaggregation and hyperplasia were found in the deprived medium (using PBS instead of enriched medium to stimulate a stressful cytoplasmatic gradient) cultures even for the WT cells (Figure 2B). Cell hyperplasia and atypical morphology was also observed in ATP4A mutated PCs [14]. Thus, cell KOs for the SLC26A7, SLC26A9 and SLC4A2 genes could reproduce the acid –base balance malfunction previously observed for the ATP4A mutated patients, which affected the mitochondrial gradient and triggered ROS damage.

A viability study was evaluated by TMRE fluorescence staining and measured using flow cytometry. WT HEK293T cells, apoptosis induced-cells (positive control, C+) and KO cells cultured in both enriched (E+) and restrictive (R-) media were included (Figure 2C). A significant decreasing viability was observed in both WT and C+ cells when compared to E+ and R- medium cultures. However, no differences were observed for KO cell viability between the E+ and R- media. Finally, different viability percentages were observed between WT and C+ cells compared to KO cell lines. Our results suggested that there was no cytoplasmatic gradient stress affecting KO cell lines compared to the WT or C+ cultures. A cell cycle phase analysis was calculated for the apoptosis evaluation, by means of differential staining of DNA and RNA. The percentage of the SubG1 phase (apoptotic) was calculated in cells cultured in E+ and R- media (Figure 2D). No differences in the number of apoptotic cells were observed between WT and KO cells in E+ medium, while significant differences were observed when comparing cells grown in the R- medium. In order to evaluate cell activity, separation of the SubG1 phase in G0 and G1 cell cycle phases was performed by using Hoechst33342 and Pyronin Y dyes, which exclusively react with DNA or with both DNA and RNA, respectively. 59.7% and 58.5% of the WT cells were found in G1 phase in enriched and restrictive media, respectively (Figure S2B). However, the 56.6% and 62.4% KO cells were found in G1 phase in average for KO cells in enriched and restrictive media, respectively (Figure S2B). Thus, the percentage of active cells was observed to be reduced in the restrictive medium compared to the enriched medium in WT cells, while the percentage of active cells increased in the restrictive medium compared to the enriched medium in KO cells. This observation suggests that KO cells are more quiescent than WT cells in normal conditions. Interestingly, KO cells are even more active than WT cells under restrictive conditions (Figure S2B).

Finally, R- cultured cells were evaluated with DCFDA fluorescence staining (ROS damage) in order to correlate the apoptosis percentage differences with the mitochondrial function (Figure 2E). Significant fluorescence differences were observed between WT cells compared to KO cell lines, suggesting that the increased apoptosis in KO cell lines was triggered by ROS damage activated by mitochondrial damage, as previously observed for mutated ATP4A.

### 3.2. Autoimmune Polyendocrine Syndrome (APS) Series

In order to explore the prevalence of alterations in the acid–base balance mechanism that explain autoimmune gastric and thyrogastric pathologies (APS pathologies including gastric and thyroid disease) a new series of patients was recruited and studied. A new series of 69 patients including 50 familial (F8 to F57) and 19 sporadic-like (S1 to S19) patients with different co-occurring immunodeficiencies was recruited (Table 1). This series, plus the five thyrogastric families (F3 to F7) and the two previously studied F1 and F2 families were evaluated (N = 76).

#### 3.2.1. Pathology Associations

The frequency of the pathology associations was evaluated in familial and sporadic patients (Table 2). CAG and Hashimoto’s disease were the most associated diseases in both familial and sporadic patients, with similar percentages (>20% in both scenarios). Interestingly, Graves’ disease only co-occurs with CAG but not with gNETs in familial and sporadic patients. On the other hand, gNETs are strongly associated with Hashimoto’s disease in familial patients (81.8%), but this number drops to 36.4% in sporadic patients. In total, CAG (21.4%) and Hashimoto’s disease (24.8%) were the most associated with other pathologies compared to gNETs (7.0%) and Graves’ disease (4.6%), respectively, which suggests a correlation between the severity (gNETs) and an exclusive origin; conversely, CAG is frequently associated with more pathologies, which suggests a common origin for multiple co-occurring diseases. Similarly, other APS pathologies are also more associated with CAG and Hashimoto’s disease in the total series (Table 2). Interestingly, APS3 also co-occurs with APS1 and APS2 pathologies, suggesting an overlap of APS subtypes different from the current classification (Figure 1). In addition, APS3 (thyroid) and APS3B (gastric) also co-occur with DM2, which is a non-APS disease (Table 2).

Regarding the age of onset, no significant differences were found between familial and sporadic patients (Figure 2F). Only gNETs had a slightly earlier age of onset in familial patients (53.8 years) compared to sporadic patients (63.0 years), which might correlate with the later somatic components of gastric-only disease patients. Between pathologies, gNETs have a later age of onset compared to CAG in both familial and sporadic patients, which is probably because gNETs arise as a result of the CAG condition. When studied together, no significant differences in the age of onset were found between thyroid disease (39.2 years) and other immunodeficiencies (40.0 years), but both had an earlier age of onset compared to CAG (44.8 years; *p*-value = 0.043) and gNETs (56.4 years; *p*-value = 0.0012) (Figure 2F).

Regarding co-occurrence, gastric disease is frequently diagnosed as the last pathology (91%), while thyroid and other immunodeficiencies are non-differentially diagnosed as first and second pathologies within the same patient. Interestingly, the time-lapse between the age of onset is significantly higher between first and second pathologies (11.5 years) compared to that between the second and third disease (2.5 years) (*p*-value = 0.0115) (Figure 2G).

#### 3.2.2. Genetic Studies

In order to explore the genetic origin of APS pathologies and the prevalence of variants in the genes previously found in the Discovery WES1 study, we sequenced a new series of 69 patients by tNGS using a custom panel of 12 genes involved in the acid–base balance of PCs (see Materials and Methods). Forty healthy Spanish individuals were included in the tNGS study as controls. Variants were discarded when they were also found in controls. Samples from different members of 13 families were collected and partial segregation was obtained (Figure S1). We used these data in order to filter variants. Non-segregating variants found in healthy family members were discarded. Only putatively pathogenic variants were considered. In total, 64 filtered putatively damaging variants were found in 54.17% of all studied patients (N = 76) (Table S2). No variants were found in 24 of the studied patients or in three of the genes included in the panel (KCNE2, KCNQ1 and CCKBR2) (see also Table 1). The percentage of variants was calculated per allele to differentiate between heterozygous and homozygous states. Thus, variants were found in 42.1% of all alleles (Table S3). In familial patients, similar percentages were found for gNET (46.1%) and CAG (43.4%), while in sporadic patients, significantly different percentages were found for gNETS (13.6%) and CAG (43.7%) (*p* < 0.05). Our results suggest a correlation between gastric severity and positivity for the panel. Interestingly, a high percentage of variants was found for non-gastric patients (75.0%), which demonstrates that observed differences were not associated with the selected genes of the panel. The distribution of variants within genes was evaluated along genes (Figure S3). No hotspots were found within genes per total variants or associated with immunodeficiency types.

- Efficacy per patient group.

The percentage of positivity was calculated per patient group (Table 3). Almost 44% of the pathologies in familial probands were explained by variants in genes of the panel versus 26.3% in the sporadic patients (*p*-value < 0.05). No significant differences between positivity for thyroid diseases (Graves’ or Hashimoto’s) and gastric diseases (CAG or gNETs) were observed in familial patients. In sporadic patients, significant differences were not observed between thyroid diseases either, but 13.6% of gNET patients and 43.8% of CAG patients harbored variants in genes of the panel, which suggests that gastric severity might correlate with somatic components in sporadic patients. Regarding non-APS pathologies, autoinflammatory diseases have lower positivity percentages, which suggests that other mechanisms may be involved; however, 50.0% of positives were found for DM2, which suggests that this nonimmune disease might be correlated with the acid–base balance.

- Efficacy per gene.

Positivity was evaluated per gene to establish phenotype–genotype correlations. The highest percentages of positive patients were found for the genes SLC26A9 (27.5%), SLC26A7 (15.9%) and ATP4A (20.9%) (Table 3), with similar percentages for both familial and sporadic individuals (Table S4). ATP4A variants represent 46.7% of variants associated with gNETs, while in CAG they only represent 13.3%, which demonstrates not only the importance of this gene, but also its link to the severity of gastric pathology. By contrast, only variants in SLC9A2/4 and KCNQ1 were associated with CAG, which suggests that the role of these genes is less relevant in gastric pathology. In the thyroid, ATP4A and PTH2R variants were only found in Hashimoto’s disease, while SLC9A4 variants were only found in Graves’ disease. Variants in PTH1R/2R genes, which are involved in Ca^2+^ metabolism, had the highest percentages in APS3D (collagen). Variants in SLC26A9 had the highest percentage in APS3A, APS3C, DM2 and autoinflammatory diseases, which suggests highly heterogeneous pathogenic effects for variants of this gene.

- Efficacy per gene in co-occurring pathologies.

The expression of genes included in the panel was observed not only in PCs, but also in thyrocytes, enterocytes and melanocytes (Figure S4). Thus, a unique mutation in these co-expressed genes might affect different tissues simultaneously and explain the co-occurrence of autoimmune pathologies. To this end, positivity was also calculated per co-occurring pathology in order to test co-expression and paired diseases with a common genetic origin (Table 3).

The highest percentage of positivity for gastric pathologies (gNETs and CAG) co-occurring with thyroid disease, was found for Hashimoto’s disease (70.0% and 65.2%, respectively). Graves’ disease was only associated with CAG (Table 3). A high positivity for the other APSs was found when these were co-occurring with Hashimoto’s disease, in agreement with the clinical classification (Figure 1). Interestingly, a higher positivity for nonautoimmune DM2 was found when co-occurring with gNETs, as we previously observed in the pathology association studies. Similar results were observed for familial and nonfamilial patients (Table S5).

- Correlation of the efficacy with the number of pathologies per patient.

The observed high positivity percentages for gastric disease co-occurring with thyroid diseases demonstrate the relevance of the common genetic origin for thyrogastric pathology. Around 67% and 33% of thyrogastric patients with co-occurring additional APSs were positive for gNETs and CAG, respectively (Table 1). In addition, we compared the correlation between percentages of positivity with the total amount of pathologies per patient. An inverse correlation was found between patients with variants (positivity) and the number of pathologies. In patients with two, three and four pathologies, positive alleles were found in 47.8%, 41.9% and 32.1%, respectively. The same correlation was found for CAG patients: positive alleles were found in 55.8%, 36.1% and 22.7% of patients affected with two, three and four pathologies, respectively. However, a direct correlation was found for gNET patients. In gNET patients with two, three and four pathologies, positive alleles were found in 20.0%, 44.4% and 50.1%, respectively. Thus, positivity in CAG patients decreases when the number of co-occurring pathologies increases, while positivity in gNETs patients increases with the number of co-occurring pathologies.

The percentage of patients with variants was calculated in order to evaluate the genetic relevance per pathology. The highest percentage of total positive patients was associated with variants in genes involved in the acid–base balance. A positivity of 63.3% and 50% was found for variants in genes involved in the acid–base balance in gastric and thyroid diseases, respectively. By contrast, positive patients with other APSs and inflammatory disease had variants in genes mainly involved in processes other than the acid–base balance.

## 4. Discussion

A series of 76 patients affected with different autoimmune pathologies (APS) was evaluated in this study in order to identify the genetic landscape underlying the association of autoimmune pathologies in 19 sporadic and 57 familial patients. Only putatively pathogenic variants and not found in healthy controls were considered. Slight differences were found between both groups, which highlights the importance of genetic involvement in co-occurring pathologies, even when there are no familial antecedents (sporadic-like patients). We have identified a set of genes chiefly involved in the cellular acid–base balance, which would explain the co-occurrence of autoimmune pathologies under a genetic scenario or a scenario involving the alteration of downstream events. Assuming monogenic (involving genes expressed in various affected tissues) and polygenic (involving multiple tissue-specifically expressed genes) scenarios has allowed us to describe the role of genetics to redefine the APS classification established by Neufeld and Blizzard in 1980 [20] and propose a novel APS classification based on the genetic landscape that will improve diagnosis, monitoring and prevention.

### 4.1. Achlorhydria and Gastric Disease

Gastric autoimmune disease involves hypergastrinemia and PC atrophy that triggers achlorhydria and anemia [21], but because of the complex epidemiology and pathogenesis as well as the frequent overlap with the etiology of H. pylori infection, autoimmune gastritis and gNETs are not well understood [22]. A significant heritable component was demonstrated for gastric autoimmune disease, although no genetic susceptibility evidence has been described [23]. However, we recently demonstrated the relevance of mutations in the ATP4A gene in achlorhydria and its importance for CAG, gNETs and derived secondary viral infections [11,12,14]. Here, we have described four new variants in the ATP4A gene in 46.7% and 13.3% of the studied patients with gNETs and CAG, respectively, which demonstrates not only the role of this gene in gastric disease but also its correlation with disease severity. In addition, we found another seven genes that might correlate with the achlorhydria scenario and gastric disease. Five genes are involved in the regulation of the acid–base balance in PCs (SLC26A7, SLC26A9, SLC4A2, SLC9A2 and SLC9A4) and the other two genes are involved in PC function (PTH1R and PTH2R). The SLC26 anion transporter family is becoming of interest in gastrointestinal tract disease [24]. Importantly, KO mouse models for SLC26A7, SLC26A9, and SLC4A2 were described with gastric achlorhydria [17,18,19], which demonstrates that alterations found in our patients might explain gastric disease. Protein level studies in human gastric tissue were not assessed because parietal cells are atrophic, defective, hyperplastic or not present under gastritis or gNET scenarios. Thus, no precise detection of the proteins codified by our genes of interest might be detected in gastric tissue. However, our in vitro studies, demonstrated that ROS damage-mediated apoptosis, hyperplasia and altered morphology occurred in KO cell lines (Figure 2A–E). A higher viability was also found in KO cell lines when an alteration of the acid–base balance was induced (restrictive media), suggesting that KO cells might offset osmotic stress because of an altered solute transport function. Our results suggest that mutations in *SLC26A7*, *SLC26A9* and *SLC4A2* found in thyrogastric patients were involved in the same mechanism that we previously observed in ATP4A mutated patients; where alterations in the acid–base balance interfered with the function of mitochondria, which activated ROS signaling and triggered caspase-3-mediated apoptosis of parietal cells and the immune response [14]. SLC4A2 deficient mice also develop antimitochondrial antibodies [19].

### 4.2. Autoimmune Thyrogastric Syndrome

Hashimoto’s disease (hypothyroidism) is associated in 40% of patients with autoimmune gastritis and a genetic component has been confirmed when these diseases co-occur (autoimmune thyrogastric syndrome) [16]. Even though several pathogenic mechanisms have been described for autoimmune thyroid disease [25], no common etiology has been established when it is associated with autoimmune gastric disorders (autoimmune thyrogastric syndrome). Some proposed mechanisms involve complex interactions among embryological and immunological factors, but they lack sufficient robustness to firmly establish a causative relationship. At the biochemical level, some studies pointed out that gastric mucosal and thyroid follicular cells have a Na+/I− symporter in common that involves similar peroxidase enzymes (GPO and TPO, respectively) [16]. In line with this hypothesis, our findings indicate a common genetic origin for both pathologies. Cotransporter genes involved in achlorhydria (SLC26A7, SLC26A9 and ATP4A) were also strongly associated with thyroid disease in thyrogastric patients, suggesting a monogenic model for the thyrogastric syndrome (Table 3). Remarkably, SLC26A7 was found to be expressed in both gastric and thyroid tissues (Figure S4) and recently, alterations in SLC26A7 were reported associated with in thyroid diseases, such thyroid dyshormonogenesis [26], congenital goitrous hypothyroidism [27] and primary anaplastic thyroid cancer [28]. Similarly, variants in other thyroid-only expressed genes involved in the acid–base balance (not included in our panel) might compose a digenic scenario together with genes expressed in PCs.

Moreover, we found differential clinical associations for both thyroid diseases included in this study, which are important at the translational level and for follow-up recommendations. Graves’ disease was exclusively associated with CAG patients, while Hashimoto’s disease was more frequently associated with CAG (62.7%) compared to gNET patients (25.5%) (Table 2). A previous clinical evaluation of the thyrogastric syndrome observed the same association [29]. However, although this co-occurrence was the most frequent clinical co-occurrence, a previous study reported a gNET associated with Graves’ disease [30].

In summary, our results suggest that a higher gastric severity was less associated with thyroid disease. Independently of the severity, autoimmune thyrogastric syndrome would correlate with variants in genes involved in the acid-base balance, either in monogenic (co-expression) or digenic scenarios, which would trigger the mitochondrial malfunction and autoimmune response, as we previously described for achlorhydria disease [14].

### 4.3. Co-Occurring Immunopathies (Polyautoimmune Syndrome)

It is well documented that gastric autoimmune disease frequently occurs together with other autoimmune conditions. APS patients have a high prevalence of gastric disease and anemia. In addition to thyroid disease, other autoimmune diseases such as DM 1 [31], Vitiligo [32], Addison’s disease [33] and myasthenia gravis [34] typically co-occur with autoimmune gastritis. Individual autoimmune diseases are associated with defects in the immune system and HLA alleles [3]; however, although a marked genetic component was observed in patients with co-occurring pathologies, no susceptibility genes have been identified for APS [4]. Thus, understanding the genetic landscape of gastric autoimmune disease is relevant to other autoimmune diseases, given their clinical overlap.

#### 4.3.1. Pathogenic Mechanisms

Our findings suggest a strong genetic background for the co-occurrence of immunopathies, rather than it being an incidental clinical finding. The candidate genes for causing achlorhydria might play an important role in both familial and sporadic APS; this may involve the expression of achlorhydria genes in other tissues (monogenic scenario), or the accumulation of variants in tissue-specific genes together with achlorhydria genes (polygenic scenario). It is also possible that achlorhydria caused by tissue-specific gastric genes alters downstream pathways.

**Figure 3:**
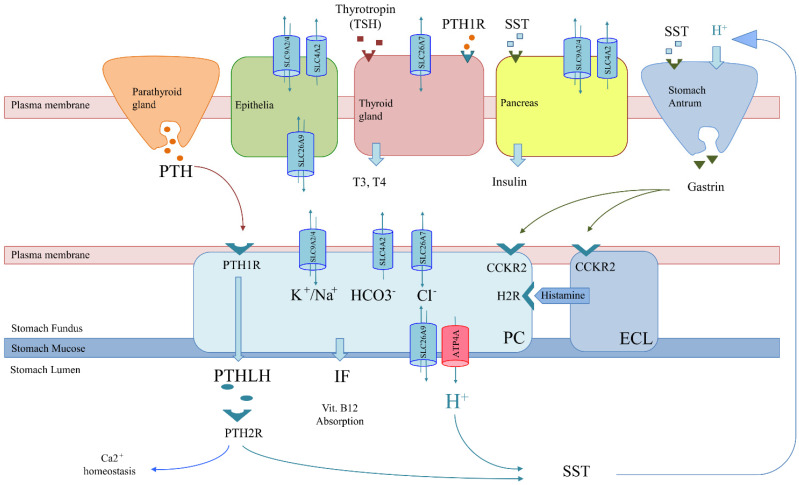
Genetics-based mechanism for co-occurring pathologies in thyrogastric and APS disease. Solute carriers (SLCs) in tissues of interest and regulation involving parietal cells (PCs) are shown: parathyroid gland (orange), melanocytes (green), thyroid gland (red), pancreas (yellow) and stomach (blue), which is composed of antrum (gastrin secretion) and fundus (PC and ECL) areas. The tissue-specifically expressed gene (ATP4A) is shown in red. More widely expressed SLCs (co-expression) with variants observed in patients of the studied series are shown in blue (SLC26A7, SLC26A9, SLC4A2, and SLC9A2/A4). The non-tissue-specific expression of SLCs correlates with a monogenic model for co-occurring pathologies, as a unique genetic variant would alter the function of different tissues simultaneously. However, co-occurrence may also be explained by a pathway alteration model. PCs (blue) export H^+^ and parathyroid hormone 1 like hormone (PTHLH) to produce gastric acid at the stomach lumen and regulate the Ca^2+^ absorption pathway, respectively. H^+^ secretion is positively regulated by gastrin. Somatostatin (SST) negatively regulates gastrin but is also involved in insulin secretion by the pancreas. Likewise, PTHLH secretion is regulated by PTH from the parathyroid gland. Thus, variants in the PTH/PTHLH pathway deregulate PC function but would also affect Ca^2+^ homeostasis associated with collagen diseases.

- Co-expression scenario: Genes involved in achlorhydria are also expressed in tissues other than gastric tissues (SLC26A7, SLC26A9, SLC4A2, SLC9A2 and SLC9A4) (Figure 3). Thus, like the thyrogastric syndrome, APS might be explained by monogenic models with variants in genes from the achlorhydria panel, since high positivity was observed in several associated immunodeficiencies (Table 3). Similarly, the co-expression scenario comprises not only SLC genes but also other genes, such as AIRE [7] and CTLA-4 [8], which were previously described to be involved in APS disorders.

- Polygenic model: The cumulative effect of variants in tissue-specifically expressed genes mainly involved in the acid–base balance process might compose a digenic/polygenic scenario together with the ATP4A gene. On the other hand, skin pathologies as well as the set of autoinflammatory diseases had the lowest percentage of variants (positivity) in the genes included in the panel (Table 1), which suggests a polygenic model or other mechanisms different from an acid–base deregulation for APS patients when they co-occur with APS3C or autoinflammatory diseases. APS including skin diseases might be better explained by variants in genes of the immune system, as previously reported [9]. In addition, previously described susceptibility genes to explain individual pathologies, such as NLRP1 and XBP1 (vitiligo) [35], or TCF7L2, ABCC8 and CAPN10 (DM2) [36], were included in this model. However, we cannot rule out a different genetic behavior for a pathology when it is co-occurring or when it is diagnosed alone. Likewise, a polygenic model also describes a syndrome that mimic APS (APS-like syndrome) caused by an accumulation of independent pathologies with no shared genetic origin.

- Genes involved in achlorhydria that are not expressed in other tissues might interfere with downstream pathways or acid-dependent absorption. Thus, an alteration in the H+/somatostatin (SST) pathway, which is involved in thyroid regulation and gastrin and insulin secretion, may trigger not only achlorhydria but also hypothyroidism and diabetes (Figure 3). The inflammatory bowel disease described in the F1 family affected with gNETs caused by ATP4A mutations fits in this scenario [15]. Similarly, an alteration in the PTH/PTHLH pathway, which depends on PTH secreted by the parathyroid gland, would affect both gastric and Ca^2+^ homeostasis (Figure 3). The rheumatoid arthritis and gNET described in the F2 family is in agreement with this scenario [12].

#### 4.3.2. Clinical Associations

Thyroid disease was the pathology most associated with other type 3 APSs in our series of patients (Table 2), which is in agreement with the current APS classification (Figure 1) [3]. However, these associations were different for Hashimoto’s and Graves’ disease patients (Table 2). On the other hand, early onset immunodeficiencies and thyroid disease were associated with gastric disease and DM1 of late onset (Table 2). In addition, Graves’ disease only co-occurs with CAG disease (SLC9A2/4 and KCNQ1genes).

Besides, nonautoimmune pathologies are typically associated with a polygenic scenario, but none are included in the APS classification (Figure 1) [4]. Our results suggest that nonautoimmune pathologies (autoinflammatory diseases and DM2) should also be genetically associated with APS. However, low positivity percentages for genes included in the panel were found for most autoinflammatory diseases in our series of patients (Table 3), which suggests the involvement of biological processes not related to achlorhydria. Thus, autoinflammatory disease in APS patients might be compatible with polygenic models, as previously described. However, a surprisingly high positive percentage was found in the tNGS study for DM2 in familial patients (50%), which suggests a correlation with an achlorhydria-mediated gastric disease (Table 1 and Table 2) and might be explained by achlorhydria-mediated SST-deregulation (Figure 3). Interestingly, DM2 positivity contrasts with DM1, where variants are found in genes that do not express in the pancreas (ATP4A, SLC26A7 and PTH1R). This might explain the different etiology of both types of diabetes (Figure 3). In addition, DM2 remission after bariatric surgery has been widely replicated together with the observation that bariatric surgery prevents or delays incident DM2 [37], which might be correlated with a stomach SST secretion mechanism. 

Thus, DM and thyroid pathologies might serve as biomarkers for the prediction of gastric severity. In this regard, patients affected with thyroid disease plus a second immunodeficiency must alert clinicians, who should make these patient undergo a gastroenterological follow-up and perform genetic studies to link the origin of co-occurring immunodeficiencies to achlorhydria for the prevention of gastric disease. Adequate and preventive treatment for achlorhydria might prevent derived immunodeficiencies and gastric disease, as we previously described for the ATP4A mouse model [13].

## 5. Conclusions

In conclusion, here we are describing a novel genetic landscape not only for achlorhydria-mediated gastric disease, but also for autoimmune thyrogastric and APS syndromes. Altered genes will deregulate the cellular acid–base balance, affecting mitochondrial biogenesis as we previously described [14]. The proposed genetic (monogenic and polygenic) and regulation models compose a new scenario for the co-occurrence of autoimmune pathologies. In addition, our studies open a new window for prediction, monitoring and diagnosis. Surprisingly, APS1 and APS2 patients were positive for genes of the panel, diluting the relationship of the proposed mechanism with only the APS3 or thyrogastric type (Figure 1). Thus, new acid–base mechanism genes are expected to be involved for monogenic/polygenic models even with no gastric disease patients.

The current clinical classification does not assist the patient’s needs. Thus, new functional and genetic studies of different APS patients must be performed to enlarge and complete the full spectrum of susceptibility genes that will lead to a definitive APS classification that can be translated to the clinic and provide guidance for the early diagnosis, prevention and treatment of autoimmune pathologies. Understanding the genetic bases of co-concurrency will serve to anticipate diseases and to evaluate the risk of secondary pathologies.

## Figures and Tables

**Table 1:** Summary of clinical information and targeted Next Generation Sequencing (tNGS) results for the recruited patients. Previously studied patients F1 and F2 (Calvete et al. 2015 and 2017) and the 5 thyrogastric families from the Discovery WES 1 study are also shown. gNET: gastric neuroendocrine tumor; CAG: chronic atrophic gastritis.

				Clinical Information												tNGS Study					
Patients	Gastric Disease				Autoimmune Polyendocrine Syndrome (APS)						NON-APS										
		N	APS1	APS2	APS3 (Graves)	APS3 (Hashimoto)	APS3A (DM1)	APS3B (gastric)	APS3C (skin)	APS3D (collagen)	DM2	Autoinfl.	ATP4A	SLC26A9	SLC9A4	SLC9A2	PTH2R	PTH1R	SLC4A2	SLC26A7	KCNQ1
**Previously**	gNET	F1											p.Arg703Cys								
**studied**		F2											p.Gln680Leu					p.Glu546Lys			
**Discovery WES1**	gNET	F3															p.Ile194Met		p.Arg25Gln		
	CAG	F4												p.Val172Met/p.Arg849Gln							
		F5																		p.Ile215Val	
		F6											p.Pro240His							p.Ile215Val	
		F7												p.Arg849Gln							
**FAMILIAL**	gNET	F8											p.Gln680Leu/c.1500+5G>T								
		F9											p.Thr628Lys						p.Pro102Leu		
		F10											p.Pro240His								
		F11																			p.Thr312Ile
		F12												p.Ala820Ser							
		F13												p.Gly841Glu							
		F14																			
		F15																			
		F16																			
		F17																			
	CAG	F18															p.V371A*54	p.Glu546Lys			
		F19											c.1500+5G>T				p.Arg143Cys		p.Ala459Val		
		F20																c.544-6G>C			p.Val648Ile
		F21												p.Ter888GlnextTer2	p.Gly116Ser						
		F22												p.His748Arg			p.Arg143Cys			p.Glu485Val	
		F23												p.Asn501Se				p.Glu546Lys		p.Glu485Val	
		F24												p.Ala820Ser/p.Val622Leu						p.Ile215Val	
		F25											p.Arg668Cys							c.1777-4C>T	
		F26											c.1500+5G>T								
		F27																	p.Ala459Val		
		F28																p.Gly562Arg			
		F29											p.Pro240His								
		F30												p.Val744Met							
		F31																	p.Ala459Val		
		F32														p.Arg656Gln					
		F33																			
		F34																			
		F35																			
		F36																			
		F37																			
		F38																			
		F39																			
		F40																			
		F41																			
		F42																			
		F43																			
		F44																			
		F45																			
		F46																			
		F47																			
		F48																			
		F49																			
		F50																			
		F51																			
	Non-gastric	F52												p.Val172Met						p.Leu349Phe	
		F53												p.Asn501Ser				p.Glu546Lys		p.Glu485Val	
		F54											c.1500+5G>T								
		F55												p.Arg849Gln							
		F56																			
		F57																			
SPORADIC	gNET	S1																		p.Ile215Val	
		S2																		c.1777-4C>T	
		S3												p.Val172Met							
		S4																			
		S5																			
		S6																			
		S7																			
		S8																			
		S9																			
		S10																			
		S11																			
	CAG	S12															p.Arg143Cys			p.Val455Met	
		S13																	p.Pro114Leu		
		S14											c.1500+5G>T	p.Val172Met				p.Glu546Lys			
		S15																c.313+4C>T			
		S16																			
		S17																			
		S18																			
		S19																			
	Pathology found in the proband and other family members																				
	Pathology found only in the proband																				
	Pathology not found in proband but in other members of the family																				

**Table 2:** Association between pathologies (co-occurrence) in familial and sporadic cases and the total series. Relevant aspects cited in the text are highlighted in grey.

Pathologies										Autoimmune Polyendocrine Syndrome (APS)													NON-APS				
		APS1 *		APS2 *		APS3 (Graves)		APS3 (Hashimoto)		APS3A (DM1)		APS3B (gNET)		APS3B (CAG)		APS3C (skin)		APS3D (collagen)		non-gastric **		DM2		Autoinflammatory		TOTAL	
Familial	N	N	(%)	N	(%)	N	(%)	N	(%)	N	(%)	N	(%)	N	(%)	N	(%)	N	(%)	N	(%)	N	(%)	N	(%)	N	(%)
APS1 *	3	NA	NA	0	0.0	0	0.0	2	66.7	1	33.3	3	100.0	0	0.0	0	0.0	0	0.0	0	0.0	0	0.0	0	0.0	**6**	**1.7**
APS2 *	1	0	0.0	NA	NA	0	0.0	1	100.0	1	100.0	0	0.0	1	100.0	0	0.0	0	0.0	0	0.0	0	0.0	0	0.0	**3**	**0.9**
APS3 (Graves)	9	0	0.0	0	0.0	NA	NA	NA	NA	1	11.1	0	0.0	9	100.0	1	11.1	1	11.1	0	0.0	1	11.1	1	11.1	**14**	**4.1**
APS3 (Hashimoto)	43	2	4.6	1	2.3	NA	NA	NA	NA	6	13.9	9	20.9	28	65.1	13	30.2	7	16.3	6	13.9	7	16.3	15	34.9	**88**	**25.7**
APS3A (DM1)	8	1	12.5	1	12.5	1	12.5	6	75.0	NA	NA	1	12.5	6	75.0	3	37.5	0	0.0	1	12.5	0	0.0	0	0.0	**19**	**5.6**
APS 3B (gNETs)	11	3	27.3	0	0.0	0	0.0	9	81.8	1	9.1	NA	NA	NA	NA	1	9.1	1	9.1	NA	NA	4	36.36	3	27.3	**22**	**6.4**
APS 3B (CAG)	38	0	0.0	1	2.6	9	23.7	28	73.7	6	15.8	NA	NA	NA	NA	10	26.3	5	13.2	NA	NA	5	13.2	9	23.7	**73**	**21.3**
APS3C (skin)	15	0	0.0	0	0.0	1	6.7	13	86.7	3	20.0	1	6.7	10	66.7	NA	NA	3	20.0	4	26.7	2	13.3	2	13.3	**35**	**10.2**
APS3D (collagen)	9	0	0.0	0	0.0	1	11.1	7	77.8	0	0.0	1	11.1	5	55.6	3	33.3	NA	NA	3	33.3	0	0.0	6	66.7	**23**	**6.7**
DM2	9	0	0.0	0	0.0	1	11.1	7	77.8	0	0.0	4	44.4	5	55.6	2	22.2	0	0.0	0	0.0	NA	NA	2	22.2	**21**	**6.1**
Autoinflammatory	16	0	0.0	0	0.0	1	6.2	15	93.7	0	0.0	3	18.7	9	56.2	2	12.5	6	37.5	4	25.0	2	12.5	NA	NA	**38**	**11.1**
APS (non-gastric)	6	0	0.0	0	0.0	0	0.0	6	100.0	1	16.7	NA	NA	NA	NA	4	66.7	3	50.0	NA	NA	0	0.0	4	66.7	**18**	*
**TOTAL**	**162**	**6**		**3**		**14**		**88**		**19**		**22**		**73**		**35**		**23**		**18**	*	**21**		**38**		**342**	
		APS1 *		APS2 *		APS3 (Graves)		APS3 (Hashimoto)		APS3A (DM1)		APS3B (gNET)		APS3B (CAG)		APS3C (skin)		APS3D (collagen)		non-gastric **		DM2		Autoinflammatory		**TOTAL**	
**SPORADIC**	**N**	N	(%)	N	(%)	N	(%)	N	(%)	N	(%)	N	(%)	N	(%)	N	(%)	N	(%)	N	(%)	N	(%)	N	(%)	**N**	**(%)**
APS1 *	0	NA	NA	0	0.0	0	0.0	0	0.0	0	0.0	0	0.0	0	0.0	0	0.0	0	0.0	0	0.0	0	0.0	0	0.0	**0**	**0.0**
APS2 *	1	0	0.0	NA	NA	0	0.0	1	100.0	0	0.0	1	100.0	0	0.0	0	0.0	0	0.0	0	0.0	0	0.0	0	0.0	**2**	**2.9**
APS3 (Graves)	3	0	0.0	0	0.0	NA	NA	NA	NA	0	0.0	0	0.0	3	100.0	0	0.0	0	0.0	0	0.0	1	33.3	1	33.3	**5**	**7.1**
APS3 (Hashimoto)	8	0	0.0	1	12.5	NA	NA	NA	NA	2	25.0	4	50.0	4	50.0	0	0.0	0	0.0	0	0.0	2	25.0	1	12.5	**14**	**20.0**
APS3A (DM1)	2	0	0.0	0	0.0	0	0.0	2	100.0	NA	NA	0	0.0	2	100.0	0	0.0	0	0.0	0	0.0	1	50.0	1	50.0	**6**	**8.6**
APS 3B (gNETs)	11	0	0.0	1	9.1	0	0.0	4	36.4	0	0.0	NA	NA	NA	NA	0	0.0	0	0.0	NA	NA	1	9.1	1	9.1	**7**	**10.0**
APS 3B (CAG)	8	0	0.0	0	0.0	3	37.5	4	50.0	2	25.0	NA	NA	NA	NA	1	12.5	0	0.0	NA	NA	3	37.5	2	25.0	**15**	**21.4**
APS3C (skin)	1	0	0.0	0	0.0	0	0.0	0	0.0	0	0.0	0	0.0	1	100.0	NA	NA	0	0.0	0	0.0	0	0.0	0	0.0	**1**	**1.4**
APS3D (collagen)	0	0	0.0	0	0.0	0	0.0	0	0.0	0	0.0	0	0.0	0	0.0	0	0.0	NA	NA	0	0.0	0	0.0	0	0.0	**0**	**0.0**
DM2	4	0	0.0	0	0.0	1	25.00	2	50.0	1	25.0	1	25.0	3	75.0	0	0.0	0	0.0	0	0.0	NA	NA	3	75.0	**11**	**15.7**
Autoinflammatory	3	0	0.0	0	0.0	1	33.3	1	33.3	1	33.3	1	33.3	2	66.7	0	0.0	0	0.0	0	0.0	3	100.0	NA	NA	**9**	**12.9**
APS (non-gastric)	0	0	0.0	0	0.0	0	0.0	0	0.0	0	0.0	NA	NA	NA	NA	0	0.0	0	0.0	NA	NA	0	0.0	0	0.0	**0**	*
**TOTAL**	**41**	**0**		**2**		**5**		**14**		**6**		**7**		**15**		**1**		**0**		**0**	*	**11**		**9**		**70**	
		APS1 *		APS2 *		APS3 (Graves)		APS3 (Hashimoto)		APS3A (DM1)		APS3B (gNET)		APS3B (CAG)		APS3C (skin)		APS3D (collagen)		non-gastric **		DM2		Autoinflammatory		**TOTAL**	
**TOTAL SERIES**	N	N	(%)	N	(%)	N	(%)	N	(%)	N	(%)	N	(%)	N	(%)	N	(%)	N	(%)	N	(%)	N	(%)	N	(%)	**N**	**(%)**
APS1 *	3	NA	NA	0	0.0	0	0.0	2	66.7	1	33.3	3	100.0	0	0.0	0	0.0	0	0.0	0	0.0	0	0.0	0	0.0	**6**	**1.5**
APS2 *	2	0	0.0	NA	NA	0	0.0	2	100.0	1	50.0	1	50.0	1	50.0	0	0.0	0	0.0	0	0.0	0	0.0	0	0.0	**5**	**1.2**
APS3 (Graves)	12	0	0.0	0	0.0	NA	NA	NA	NA	1	8.3	0	0.0	12	100.0	1	8.3	1	8.3	0	0.0	2	16.7	2	16.7	**19**	**4.6**
APS3 (Hashimoto)	51	2	3.9	2	3.9	NA	NA	NA	NA	8	15.7	13	25.5	32	62.7	13	25.5	7	13.7	6	11.8	9	17.6	16	31.4	**102**	**24.8**
APS3A (DM1)	10	1	10.0	1	10.0	1	10.0	8	80.0	NA	NA	1	10.0	8	80.0	3	30.0	0	0.0	1	10.0	1	10.0	1	10.0	**25**	**6.1**
APS 3B (gNETs)	22	3	13.6	1	4.5	0	0.0	13	59.1	1	4.5	NA	NA	NA	NA	1	4.5	1	4.5	NA	NA	5	22.7	4	18.2	**29**	**7.0**
APS 3B (CAG)	46	0	0.0	1	2.2	12	26.1	32	69.6	8	17.4	NA	NA	NA	NA	11	23.9	5	10.9	NA	NA	8	17.4	11	23.9	**88**	**21.4**
APS3C (skin)	16	0	0.0	0	0.0	1	6.2	13	81.2	3	18.7	1	6.2	11	68.7	NA	NA	3	18.5	4	25.0	2	12.5	2	12.5	**36**	**8.7**
APS3D (collagen)	9	0	0.0	0	0.0	1	11.11	7	77.8	0	0.0	1	11.1	5	55.6	3	33.3	NA	NA	3	33.3	0	0.0	6	66.7	**23**	**5.6**
DM2	13	0	0.0	0	0.0	2	15.4	9	69.2	1	7.7	5	38.5	8	61.5	2	15.4	0	0.0	0	0.0	NA	NA	5	38.5	**32**	**7.8**
Autoinflammatory	19	0	0.0	0	0.0	2	10.5	16	84.2	1	5.3	4	21.0	11	57.9	2	10.5	6	31.6	4	21.0	5	26.3	NA	NA	**47**	**11.4**
APS (non-gastric)	6	0	0.0	0	0.0	0	0.0	6	100.0	1	16.7	NA	NA	NA	NA	4	66.7	3	50.0	NA	NA	0	0.0	4	66.7	**18**	*
**TOTAL**	**203**	**6**		**5**		**19**		**102**		**25**		**29**		**88**		**36**		**23**		**18**	*	**32**		**47**		**412**	

NA: not applicable; * APS1 and APS2 are presented but not considered for discussion due to the low number of patients. ** Associations of non-gastric patients were not considered for the total associations.

**Table 3:** Efficacy (Positivity) per patient group; per gene; and per associated pathology. Relevant aspects cited in the text are highlighted in grey.

	Total Alleles			Autoimmune Polyendocrine Syndrome (APS)																														NON-APS						TOTAL		
				APS1 *			APS2 *			APS3 (Graves)			APS3 (Hashimoto)			APS3A (DM1)			APS3B (gNET)			APS3B (CAG)			APS3C (skin)			APS3D (collagen)			Non-gastric			DM2			Autoinfl.					
**Patients**	N	P	(%)	N	P	(%)	N	P	(%)	N	P	(%)	N	P	(%)	N	P	(%)	N	P	(%)	N	P	(%)	N	P	(%)	N	P	(%)	N	P	(%)	N	P	(%)	N	P	(%)	N	P	(%)
Familial	110	48	43.6	6	3	50.0	2	1	50.0	18	8	44.4	86	36	41.9	16	6	37.5	22	10	45.5	76	30	39.5	30	16	53.3	20	8	40.0	12	8	66.7	18	9	50.0	32	8	25.0	**338**	**143**	**42.3**
Sporadic	38	10	26.3	0	0	NA	2	1	50.0	6	3	50.0	14	5	35.7	4	2	50.0	22	3	13.6	16	7	43.8	2	0	0.0	0	0	NA	0	0	NA	6	0	0.0	6	0	0.0	**78**	**21**	**26.9**
TOTAL	148	58	39.2	6	3	50.0	4	2	50.0	24	11	45.8	100	41	41.0	20	8	40.0	44	13	29.5	92	37	40.2	32	16	50.0	20	8	40.0	12	8	66.7	24	9	37.5	38	8	21.1	**416**	**164**	**39.4**
	**Total Variants**			APS1 *			APS2 *			APS3 (Graves)			APS3 (Hashimoto)			APS3A (DM1)			APS3B (gNET)			APS3B (CAG)			APS3C (skin)			APS3D (collagen)			Non-gastric			DM2			Autoinfl.			**TOTAL**		
**Genes**	**N**		**(%)**	P		(%)	P		(%)	P		(%)	P		(%)	P		(%)	P		(%)	P		(%)	P		(%)	P		(%)	P		(%)	P		(%)	P		(%)	**P**		**(%)**
*SLC26A9*	16		24.6	1		33.3	1		50.0	3		27.3	12		25.5	3		33.3	2		13.3	11		24.4	7		38.9	2		16.7	3		42.9	5		50.0	3		30.0	**50**		**27.5**
*SLC9A4*	2		3.1	0		0.0	0		0.0	2		18.2	0		0.0	0		0.0	0		0.0	2		4.4	0		0.0	0		0.0	0		0.0	2		20.0	0		0.0	**6**		**3.3**
*SLC9A2*	1		1.5	0		0.0	0		0.0	0		0.0	1		2.1	0		0.0	0		0.0	1		2.2	0		0.0	0		0.0	0		0.0	0		0.0	0		0.0	**2**		**1.1**
*PTH1R*	8		12.3	0		0.0	0		0.0	3		27.3	5		10.6	2		22.2	1		6.7	6		13.3	2		11.1	2		16.7	1		14.3	0		0.0	1		10.0	**22**		**12.1**
*PTH2R*	5		7.7	1		33.3	0		0.0	0		0.0	4		8.5	0		0.0	1		6.7	4		8.9	1		5.6	2		16.7	0		0.0	0		0.0	1		10.0	**14**		**7.7**
*SLC4A2*	6		9.2	1		33.3	0		0.0	1		9.1	5		10.6	0		0.0	2		13.3	4		8.9	0		0.0	1		8.3	0		0.0	1		10.0	1		10.0	**16**		**8.8**
*SLC26A7*	11		16.9	0		0.0	0		0.0	1		9.1	8		17.0	1		11.1	2		13.3	9		20.0	5		27.8	2		16.7	2		28.6	0		0.0	1		10.0	**29**		**15.9**
*KCNQ1*	2		3.1	0		0.0	0		0.0	1		9.1	1		2.1	0		0.0	0		0.0	2		4.4	0		0.0	0		0.0	0		0.0	0		0.0	1		10.0	**5**		**2.7**
*ATP4A*	14		21.5	0		0.0	1		50.0	0		0.0	11		23.4	3		33.3	7		46.7	6		13.3	3		16.7	3		25.0	1		14.3	2		20.0	2		20.0	**38**		**20.9**
TOTAL	65			3		4.6	2		3.1	11		16.9	47		72.3	9		13.8	15		23.1	45		69.2	18		27.7	12		18.5	7		10.8	10		15.4	10		15.4	**182**		
**Associated**	**Total**			APS1 *			APS2 *			APS3 (Graves)			APS3 (Hashimoto)			APS3A (DM1)			APS3B (gNET)			APS3B (CAG)			APS3C (skin)			APS3D (collagen)			Non-gastric			DM2			Autoinfl.			**TOTAL**		
**Pathologies**	**N**		**(%)**	P		(%)	P		(%)	P		(%)	P		(%)	P		(%)	P		(%)	P		(%)	P		(%)	P		(%)	P		(%)	P		(%)	P		(%)	**P**		**(%)**
APS1 *	2		2.0	NA		NA	0		0.0	0		0.0	1		50.0	1		50.0	2		100.0	0		0.0	0		0.0	0		0.0	0		0.0	0		0.0	0		0.0	**4**		**2.2**
APS2 *	2		2.0	0		0.0	NA		NA	0		0.0	2		100.0	1		50.0	1		50.0	1		50.0	0		0.0	0		0.0	0		0.0	0		0.0	0		0.0	**5**		**2.7**
(Graves)	7		6.9	0		0.0	0		0.0	NA		NA	NA		NA	1		14.3	0		0.0	7		100.0	0		0.0	0		0.0	0		0.0	1		14.3	0		0.0	**9**		**4.8**
(Hashimoto)	27		26.7	1		3.7	2		7.4	NA		NA	NA		NA	4		14.8	7		25.9	15		55.6	8		29.6	3		11.1	5		18.5	4		14.8	5		18.5	**49**		**26.3**
APS3A	6		5.9	1		16.7	1		16.7	1		16.7	4		66.7	NA		NA	1		16.7	4		66.7	1		16.7	0		0.0	1		16.7	0		0.0	0		0.0	**13**		**7.0**
(gNETs)	10		9.9	2		20.0	1		10.0	0		0.0	7		70.0	1		10.0	NA		NA	NA		NA	1		10.0	0		0.0	NA		NA	3		30.0	1		10.0	**16**		**8.6**
(CAG)	23		22.8	0		0.0	1		4.3	7		30.4	15		65.2	4		17.4	NA		NA	NA		NA	4		17.4	2		8.7	NA		NA	3		13.0	1		4.3	**37**		**19.9**
APS3C	9		8.9	0		0.0	0		0.0	0		0.0	8		88.9	1		11.1	1		11.1	4		44.4	NA		NA	2		22.2	4		44.4	1		11.1	2		22.2	**19**		**10.2**
APS3D	4		4.0	0		0.0	0		0.0	0		0.0	3		75.0	0		0.0	0		0.0	2		50.0	2		50.0	NA		NA	2		50.0	0		0.0	3		75.0	**10**		**5.4**
DM II	6		5.9	0		0.0	0		0.0	1		16.7	4		66.7	0		0.0	3		50.0	3		50.0	1		16.7	0		0.0	0		0.0	NA		NA	0		0.0	**12**		**6.5**
Autoinfl.	5		5.0	0		0.0	0		0.0	0		0.0	5		100.0	0		0.0	1		20.0	1		20.0	2		40.0	3		60.0	3		60.0	0		0.0	NA		NA	**12**		**6.5**
non-gastric **	5			0		0.0	0		0.0	0		0.0	5		100.0	1		20.0	NA		NA	NA		NA	4		80.0	2		40.0	NA		NA	0		0.0	3		60.0	**15**		**8.1**
**TOTAL**	**101**			**4**			**5**			**9**			**49**			**13**			**16**			**37**			**19**			**10**			**15**			**12**			**12**			**186**		

P: Positivity; NA: not applicable. * APS1 and APS2 are presented but not considered for discussion due to the low number of patients. ** Associations of non-gastric patients were not considered for the total associations.

## Data Availability

Whole exome sequencing data have been deposited in the RD-Connect GPAP platform from the CNAG (National Center for Genomic Analysis), available at https://platform.rd-connect.eu/genomics/ (accessed on 15 November 2021, project number 634935 included in the 2017 BBMRI-LPC Whole Exome Sequencing Call).

## Supplementary Materials

The following are available online at https://www.mdpi.com/article/10.3390/cells10123500/s1, Supplementary Materials, Figure S1: Segregation studies for variants found in the genetic studies, Figure S2: RT-PCR for SLC26A7, SLC26A9 and SLAC4A2, Figure S3: Distribution of the variants found in genes from the custom panel used in the tNGS study, Figure S4: Co-expression of studied genes, Table S1: List of used primers and sequences, Table S2: List of variants found in Discovery WES1 and tNGS studies for recruited patients, Table S3: Summary of total filtered variants per panel gene in familial and sporadic APS patients subdivided per gastric disease, Table S4: Positivity per gene for familial and sporadic patients. Relevant aspects cited in the text are highlighted in grey, Table S5: Positivity per associated pathology for familial and sporadic patients. Relevant aspects cited in the text are highlighted in grey.
