# Supplementary material for: Alterations in SLC4A2, SLC26A7 and SLC26A9 Drive Acid–Base Imbalance in Gastric Neuroendocrine Tumors and Uncover a Novel Mechanism for a Co-Occurring Polyautoimmune Scenario
Source: Cells. 2021 Dec 10;10(12):3500. doi: 10.3390/cells10123500 (PMC8700745; doi:10.3390/cells10123500)
Supplement: Supplementary file 1 [file cells-10-03500-s001.zip › Supplemantary Figure.pdf]

Supplementary Figure

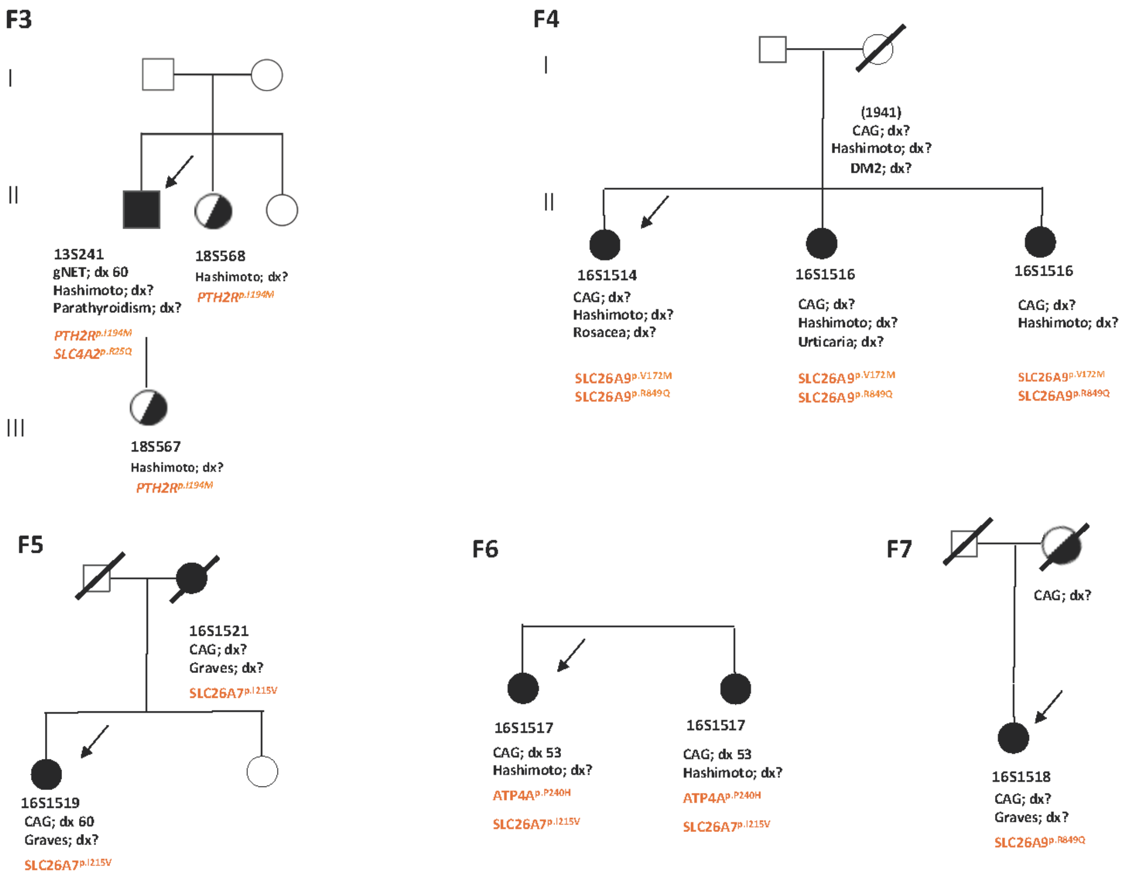

Figure S1. Segregation studies for variants found in the genetic studies. Variants are shown in orange for families F3, F4, F5, F6 and F7 (Discovery WES1 study) or in red for families F8, F11, F13, F20, F22, F23, F24 and F31 (tNGS). Non-segregating discarded variants are shown in blue. Available samples from patients included in the segregation studies are numbered. Proband included in the genetic study is indicated with a black arrow. Age at onset (dx) is shown per pathology; 'dx?' indicates unknown dx. AR: Rheumatoid arthritis. DM: diabetes mellitus. MN: multinodular.

A

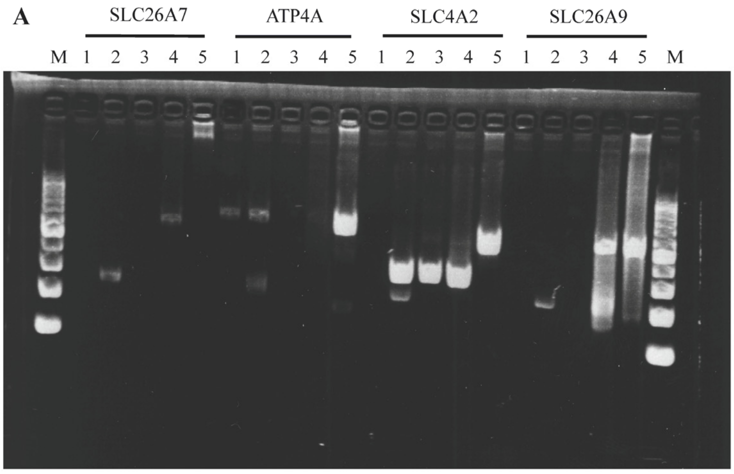

B

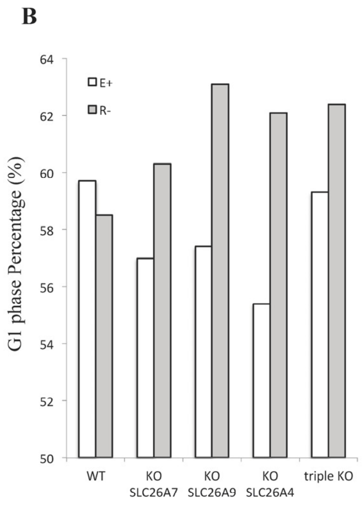

Legend

M: Marker
1. White PCR
2. HEK293 cDNA
3. NTHY cDNA
4. CAPAN1 cDNA
5. gDNA

Product sizes (pb)

Gene	cDNA	gDNA
SLC26A7	517	44816
ATP4A	311	600
SLC4A2	401	506
SLC26A9	523	523

Figure S2. (**A**) Non-cropped gel for RT- PCR of thyrogastric genes (*SLC26A7, SLC26A9, SLC4A2 and ATP4A)* by using sDNA from three different cell lines: HEK293 (renal), NTHY (thyroid) and CAPAN1 (pancreas). White PCR and genomic PCR are also showed. Expected size of PCR products for cDNA and gDNA are showed in pair bases (pb). (**B**) Percentage (%) of WT HEK293T cells and KO cells activity (G1 phase). Cells grown in enriched (E+) and restrictive (R-) mediums were included in the study.

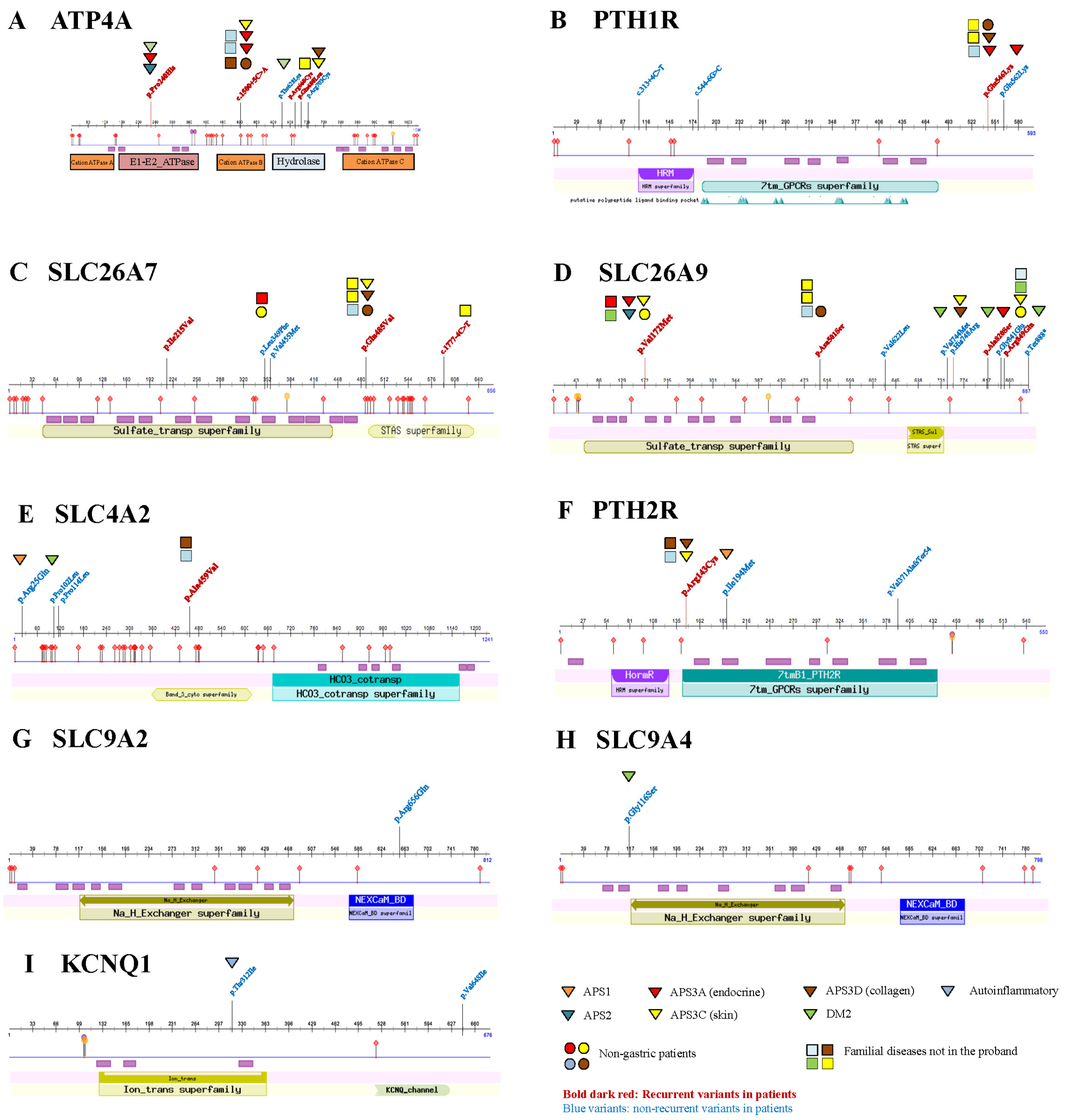

Figure S3. Distribution of the variants found in genes from the custom panel used in the tNGS study. No variants were found in the genes *KCNE2*, *KCNQ1* and *CCKBR2*. Recurrent variants and variants found in only one patient are shown in dark red and blue, respectively. Immunodeficiencies of the patient carrying the annotated variant are also indicated.

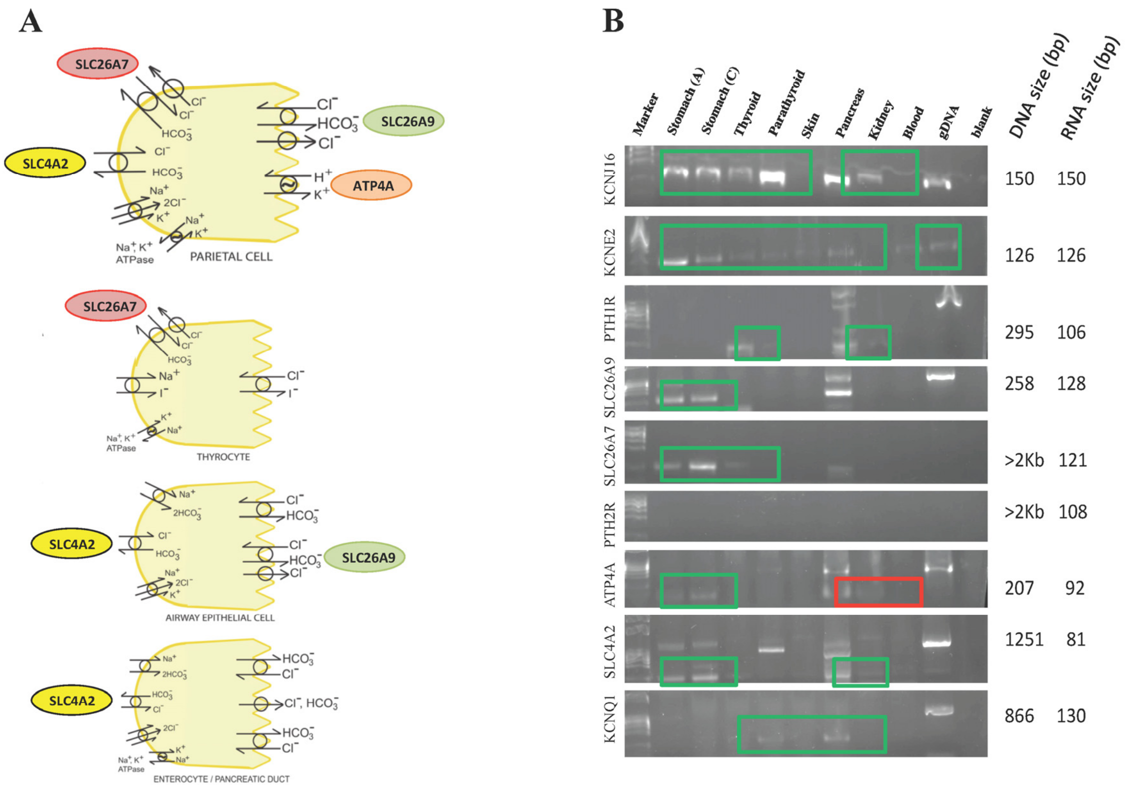

Figure S4. Co-expression of studied genes. (**A**) Co-expression of SLC genes with variants found in the Discovery WES1 study. *ATP4A* only expresses in the PCs of the stomach. However, expression of *SLC26A7*, *SLC26A9* and *SLC4A2* not only occurs in gastric tissue, but also contributes to regulating the acid-base balance or the export/import function in thyrocytes (*SLC26A7*), airway epithelial cells (*SLC4A2* and *SLC26A9*) and enterocytes (*SLC26A9*). (**B**) Co-expression of genes included in the custom panel for tNGS studies. Qualitative RNA expression was tested in normal human tissues of interest. Antrum (A) and Fundus/Corpus (C) regions of the stomach were included. Heterogeneous RNA was obtained for pancreas and skin tissues due to the large number of different cell types and keratinization, respectively. *ATP4A* and *SLC26A9* only expressed in gastric tissue and *SLC26A7* and *SLC4A2* expression was found in gastric and thyroid tissues, and gastric and pancreas tissues, respectively. *PTH1R* expressed in thyroid and pancreas tissue, while no expression was found for PTH2R in any studied tissue Almost ubiquitous expression was found for the genes *KCNJ16*, *KCNE2* and *KCNQ1*. Genomic DNA from peripheral blood was included as control. Sizes of both mRNA and DNA expected products are shown. Green squares indicate expected size-amplified product. Red square indicates an artifact product of the ATPase amplification own of the studied tissue of different than *ATP4A*.
