# Supplementary material for: Alterations in SLC4A2, SLC26A7 and SLC26A9 Drive Acid–Base Imbalance in Gastric Neuroendocrine Tumors and Uncover a Novel Mechanism for a Co-Occurring Polyautoimmune Scenario
Source: Cells. 2021 Dec 10;10(12):3500. doi: 10.3390/cells10123500 (PMC8700745; doi:10.3390/cells10123500)
Supplement: Supplementary file 1 [file cells-10-03500-s001.zip › Supplemental Table S1.pdf]

Table S1. List of used primers and sequences.

Oligosnucleotides for gene Expression (rt-PCR)		
Gene	Forward	Reverse
SLC26A7	GTGCTGGGCTTATCCGACTT	AGAGGATGTTCATCGGGGGA
SLC26A9	GTCTGGTGAGCCCACTCTTC	ATGTTTCAGGAGGCTGAGGC
SLC4A2	CAGATCAAGGCCGAGGACAG	TCTCCAGCAGTTTCAGCTCG
Oligonucleotides (cloning)		
Gene	Forward	Reverse
SLC26A7 guide 1	CACCGACAATCCGTTCCACGGCGT	AAACACGCCGTGGAACGGATTGTC
SLC26A7 guide 2	CACCGCAGAGTAACACAAGCGTGCT	AAACAGCACGCTTGTGTTACTCTGC
SLC26A9 guide 1	CACCGCTCTGTCTACCACGTAGCG	AAACCGCTACGTGGTAGACAGAGC
SLC26A9 guide 2	CACCGTCTCAAACTCATCGTCGAAG	AAACCTTCGACGATGAGTTTGAGAC
SLC4A2 guide 1	CACCGACGAACTTCACCGCACCCT	AAACAGGGTGCGGTGAAGTTCGTC
SLC4A2 guide 2	CACCGTGGAGAAACCCCGACCATTG	AAACCAATGGTCGGGGTTTCTCCAC
Oligonucleoaides for KO verification		
Gene	Forward	Reverse
SLC26A7	TGCCAATCTGCAGAGAGGTG	AAAACACCAGCTGCCTTCCC
SLC26A9	GCAGGGAGTCTGACGGAAAA	ATGGTTCTTGCTCCTGCTCC
SLC4A2	CTGGGAAGGGTGAGGTTTCG	
sgRNA sequences		
SLC26A7 (Hygromycin LV backbone)	GACAATCCGTTCCACGGCGT	TGG
	CAGAGTAACACAAGCGTGCT	GGG
SLC26A9 (Blasticidin LV backbone)	GCTCTGTCTACCACGTAGCG	GGG
	TCTCAAACTCATCGTCGAAG	AGG
SLC4A2 (Puromycin LV backbone)	GACGAACTTCACCGCACCCT	GGG
	TGGAGAAACCCCGACCATTG	AGG
