# Supplementary material for: Alterations in SLC4A2, SLC26A7 and SLC26A9 Drive Acid–Base Imbalance in Gastric Neuroendocrine Tumors and Uncover a Novel Mechanism for a Co-Occurring Polyautoimmune Scenario
Source: Cells. 2021 Dec 10;10(12):3500. doi: 10.3390/cells10123500 (PMC8700745; doi:10.3390/cells10123500)
Supplement: Supplementary file 1 [file cells-10-03500-s001.zip › Supplemental Table S2.pdf]

Table S2. List of variants found in Discovery WES1 and tNGS studies for recruited patients. Previously described variants in F1 and F2 are also shown (N=76). No variants were found in the panel genes *KCNE2*, *KCNQ1* and *CCKBR2*. Minor allele frequency (MAF) is annotated from European population in Exome Variant server from Exome Sequencing Project (ESP) database and 1000 Genomes project. Sift and Polyphen scores were considered for damage prediction annotation (D: Damaging; PD: Provably Damaging; T: Tolerated)

Gene	position	cDNA	Protein	dbSNP	ESP MAF	1000 Genomes MAF	Total	Controls	SIFT	Polyphen
SLC26A7	91334295	c.643A>G	p.Ile215Val	rs16912250	0.03047	0.0206	4*	2	T	PD
	91363504	c.1454A>T	p.Glu485Val	rs140569478	0.0	0.0024	3	0	D	PD
	91343370	c.1045C>T	p.Leu349Phe	rs113085951	0.001	0.004	1	0	D	D
	91343371	c.1363C>T	p.Val455Met	ND	.	.	1	0	.	.
	91393793	c.1777-4C>T	Uknown frameshift	rs16912805	0.0579	0.0606	2	1	.	.
SLC26A9	205915098	c.2458G>T	p.Ala820Ser	rs34309781	0.004312	0.014	2	0	T	T
	205921757	c.1864G>C	p.Val622Leu	rs34992672,BM0911223	0.0002271	0.001	1	0	D	PD
	205915034	c.2522G>A	p.Gly841Glu	ND	0	0	1	0	.	.
	205923608	c.1502A>G	p.Asn501Ser	rs148928330	0.0	0.0008	2	0	T	PD
	205931898	c.514G>A	p.Val172Met	rs146704092	0.00227	0.0036	4*	1	D	PD
	205915010	c.2546G>A	p.Arg849Gln	rs140760240	0.001135	0.0076	3*	0	T	T
	205914894	c.2662T>C	p.Ter888GlnextTer2	ND	0	0	1	0	.	.
	205918866	c.2230G>A	p.Val744Met	rs3811428,CM128010	0.001628	0.0036	1	0	D	T
	205918853	c.2243A>G	p.His748Arg	rs16856462	0.0	0.0763	1	0	T	PD
SLC4A2	151070273	c.1376C>T	p.Ala459Val	rs140685664	0.001163	0.001	3	0	T	PD
	151064649	c.341C>T	p.Pro114Leu	rs200121811,COSM3879367	0	0	1	0	D	PD
	151064613	c.305C>T	p.Pro102Leu	rs146857685	0.0004	0.001	1	0	T	T
	151064628	c.320G>A	p.Arg107Gln	rs145741704	0.0015	0.0013	1	0	T	D
PTH1R	46903510	c.1636G>A	p.Glu546Lys	rs77048718	0.004085	0.0086	5**	2	PD	T

	46895873	c.313+4C>T	Uknown frameshift	rs200207404	0.001047	0.0004	1	0	.	.
	46898372	c.544-6G>C	Uknown frameshift	rs764927778	0	0	1	0	.	.
	46903558	c.1684G>A	p.Gly562Arg	rs749330849,COSM730733		1		0	D	D
ATP4A	35560431	c.719C>A	p.Pro240His	rs139075511,COSM3692657	0.002724	0.0042	3*	1	D	PD
	35558357	c.1500+5G>T	Uknown frameshift	rs145047297	0.022	0.0066	5	0	.	.
	35555680	c.2002C>T	p.Arg668Cys		0.0002	0	2	0		
	35555799	c.1883C>A	p.Thr628Lys	ND	0	0	1	0	.	.
	35555558	c.2039A>T	p.Gln680Leu	rs61729956	0.0004539	0.0016	2**	0	D	PD
		G	A	c.2107 C>T	p.R703C	ND	0	0	.	.
PTH2R	208442379	c.427C>T	p.Arg143Cys	rs61744730,COSM2157069	0.003404	0.0098	3	1	D	D
	208489046	c.1112delT	p.Val371AlafsTer54	rs760717672	0	0	1	0	.	.
	.	.	p.Ile194Met	rs143633380	0	0	1*	0	D	D
SLC9A2	102704665	c.1967G>A	p.Arg656Gln	rs115244050	0.002674	0.0004	1	0	PD	T
KCNQ1	2847914	c.1942G>A	p.Val648Ile	rs34150427	0.0002367	0.0074	1	0	T	T
	2583448	c.935C>T	p.Thr312Ile	rs120074182,CM960901				1	D	D
SLC9A4	102478928	c.346G>A	p.Gly116Ser	rs17027275	0.0001163	0.0088	1	0	D	PD

* Variants found in the Discovery WES1 study

** Variants found in F2 (Calvete et al. 2017)

*** Variant found F1 (Calvete et al. 2019)
