# Supplementary material for: Alterations in SLC4A2, SLC26A7 and SLC26A9 Drive Acid–Base Imbalance in Gastric Neuroendocrine Tumors and Uncover a Novel Mechanism for a Co-Occurring Polyautoimmune Scenario
Source: Cells. 2021 Dec 10;10(12):3500. doi: 10.3390/cells10123500 (PMC8700745; doi:10.3390/cells10123500)
Supplement: Supplementary file 1 [file cells-10-03500-s001.zip › Supplemental Table S3.pdf]

Table S3. Summary of total filtered variants per panel gene in familial and sporadic APS patients subdivided per gastric disease.

PATIENTS	Gastric disease	N	Alleles	PANEL GENES												Total	%
				ATP4A	*SLC26A9*	*SLC9A4*	*SLC9A2*	*PTH2R*	*PTH1R*	*SLC4A2*	*SLC26A7*	*KCNQ1*	*KCNE2*	*KCNJ16*	*CCKBR*		
**FAMILIAL**	gNET*	13	26	5	2	0	0	1	1	2	0	1	0	0	0	12	46.1
	CAG**	38	76	5	9	1	1	3	4	3	6	1	0	0	0	33	43.4
	Non-gastric	6	12	3	3	0	0	0	1	0	2	0	0	0	0	9	75.0
	Total	57	114	13	14	1	1	4	6	5	8	2	0	0	0	54	47.4
**SPORADIC**	gNET	11	22	0	1	0	0	0	0	0	2	0	0	0	0	3	13.6
	CAG	8	16	1	1	0	0	1	2	1	1	0	0	0	0	7	43.7
	Total	19	38	1	2	0	0	1	2	1	3	0	0	0	0	10	26.3
**TOTAL**	gNET	24	48	5	3	0	0	1	1	2	2	1	0	0	0	15	31.2
	CAG	46	92	6	10	1	1	4	6	4	7	1	0	0	0	40	43.5
	Non-gastric	6	12	3	3	0	0	0	1	0	2	0	0	0	0	9	75.0
	Total	76	152	14	16	1	1	5	8	6	11	2	0	0	0	64	42.1

* Includes the variants previously described in families F1 and F2 (Calvete et al. 2015 and 2017).

** Includes the variants found in the 5 thyrogastric families from the Discovery WES 1 study.
