# Supplementary material for: Alterations in SLC4A2, SLC26A7 and SLC26A9 Drive Acid–Base Imbalance in Gastric Neuroendocrine Tumors and Uncover a Novel Mechanism for a Co-Occurring Polyautoimmune Scenario
Source: Cells. 2021 Dec 10;10(12):3500. doi: 10.3390/cells10123500 (PMC8700745; doi:10.3390/cells10123500)
Supplement: Supplementary file 1 [file cells-10-03500-s001.zip › Supplemental Table S4.pdf]

Table S4. Positivity per gene for familial and sporadic patients. Relevant aspects cited in the text are highlighted in grey.

				**AUTOIMMUNE POLYENDOCRINE SYNDROME (APS)**																				**NON-APS**					
	**Panel**	**Total**		APS1*		APS2*		(Graves)		(Hashimoto)		APS3A		(gNET)		(CAG)		APS3C		APS3D		non-gastric**		DM2		Autoinfl.		**TOTAL**	
**Patients**	**Gene**	**Variants**	**(%)**	N	(%)	N	(%)	N	(%)	N	(%)	N	(%)	N	(%)	N	(%)	N	(%)	N	(%)	N	(%)	N	(%)	N	(%)	**N**	**(%)**
Familial	*SLC26A9*	14	25.5	1	33.3	0	0.0	2	25.0	10	25.0	2	33.3	2	15.4	9	25.7	7	38.9	2	16.7	3	30.0	5	50.0	3	30.0	**43**	**27.6**
	SLC9A4	2	3.6	0	0.0	0	0.0	2	25.0	0	0.0	0	0.0	0	0.0	2	5.7	0	0.0	0	0.0	0	0.0	2	20.0	0	0.0	**6**	**3.8**
	SLC9A2	1	1.8	0	0.0	0	0.0	0	0.0	1	2.5	0	0.0	0	0.0	1	2.9	0	0.0	0	0.0	0	0.0	0	0.0	0	0.0	**2**	**1.3**
	PTH1R	6	10.9	0	0.0	0	0.0	2	25.0	4	10.0	1	16.7	1	7.7	4	11.4	2	11.1	2	16.7	1	10.0	0	0.0	1	10.0	**17**	**10.9**
	PTH2R	4	7.3	1	33.3	0	0.0	0	0.0	3	7.5	0	0.0	1	7.7	3	8.6	1	5.6	2	16.7	0	0.0	0	0.0	1	10.0	**12**	**7.7**
	SLC4A2	5	9.1	1	33.3	0	0.0	0	0.0	5	12.5	0	0.0	2	15.4	3	8.6	0	0.0	1	8.3	0	0.0	1	10.0	1	10.0	**14**	**9.0**
	SLC26A7	8	14.5	0	0.0	0	0.0	1	12.5	6	15.0	1	16.7	0	0.0	6	17.1	5	27.8	2	16.7	2	20.0	0	0.0	1	10.0	**22**	**14.1**
	KCNQ1	2	3.6	0	0.0	0	0.0	1	12.5	1	2.5	0	0.0	0	0.0	2	5.7	0	0.0	0	0.0	0	0.0	0	0.0	1	10.0	**5**	**3.2**
	ATP4A	13	23.6	0	0.0	1	100.0	0	0.0	10	25.0	2	33.3	7	53.8	5	14.3	3	16.7	3	25.0	1	10.0	2	20.0	2	20.0	**35**	**22.4**
	**TOTAL**	55		3	1.9	1	0.6	8	5.1	40	25.6	6	3.8	13	8.3	35	22.4	18	11.5	12	7.7	7		10	6.4	10	6.4	**156**	
Sporadic	*SLC26A9*	2	20.0	0	0.0	1	100.0	1	33.3	2	28.6	1	33.3	0	0.0	2	20.0	0	0.0	0	0.0	0	0.0	0	0.0	0	0.0	**7**	**26.9**
	SLC9A4	0	0.0	0	0.0	0	0.0	0	0.0	0	0.0	0	0.0	0	0.0	0	0.0	0	0.0	0	0.0	0	0.0	0	0.0	0	0.0	**0**	**0.0**
	SLC9A2	0	0.0	0	0.0	0	0.0	0	0.0	0	0.0	0	0.0	0	0.0	0	0.0	0	0.0	0	0.0	0	0.0	0	0.0	0	0.0	**0**	**0.0**
	PTH1R	2	20.0	0	0.0	0	0.0	1	33.3	1	14.3	1	33.3	0	0.0	2	20.0	0	0.0	0	0.0	0	0.0	0	0.0	0	0.0	**5**	**19.2**
	PTH2R	1	10.0	0	0.0	0	0.0	0	0.0	1	14.3	0	0.0	0	0.0	1	10.0	0	0.0	0	0.0	0	0.0	0	0.0	0	0.0	**2**	**7.7**
	SLC4A2	1	10.0	0	0.0	0	0.0	1	33.3	0	0.0	0	0.0	0	0.0	1	10.0	0	0.0	0	0.0	0	0.0	0	0.0	0	0.0	**2**	**7.7**
	SLC26A7	3	30.0	0	0.0	0	0.0	0	0.0	2	28.6	0	0.0	2	100.0	3	30.0	0	0.0	0	0.0	0	0.0	0	0.0	0	0.0	**7**	**26.9**
	KCNQ1	0	0.0	0	0.0	0	0.0	0	0.0	0	0.0	0	0.0	0	0.0	0	0.0	0	0.0	0	0.0	0	0.0	0	0.0	0	0.0	**0**	**0.0**
	ATP4A	1	10.0	0	0.0	0	0.0	0	0.0	1	14.3	1	33.3	0	0.0	1	10.0	0	0.0	0	0.0	0	0.0	0	0.0	0	0.0	**3**	**11.5**
	**TOTAL**	10		0	0.0	1	3.8	3	11.5	7	26.9	3	11.5	2	7.7	10	38.5	0	0.0	0	0.0	0	0.0	0	0.0	0	0.0	**26**	

P: Positivity; NA: not applicable

*APS1 and APS2 are presented but not considered for discussion due to the low number of patients

**Associations of non-gastric patients were not considered for the total associations.
