# Supplementary material for: Alterations in SLC4A2, SLC26A7 and SLC26A9 Drive Acid–Base Imbalance in Gastric Neuroendocrine Tumors and Uncover a Novel Mechanism for a Co-Occurring Polyautoimmune Scenario
Source: Cells. 2021 Dec 10;10(12):3500. doi: 10.3390/cells10123500 (PMC8700745; doi:10.3390/cells10123500)
Supplement: Supplementary file 1 [file cells-10-03500-s001.zip › Supplemental Table S5.pdf]

Table S5. Positivity per associated pathology for familial and sporadic patients. Relevant aspects cited in the text are highlighted in grey.

				**AUTOIMMUNE POLYENDOCRINE SYNDROME (APS)**																				**NON-APS**					
		**Total pathologies**		APS1*		APS2*		(Graves)		(Hashimoto)		APS3A		(gNET)		(CAG)		APS3C		APS3D		non-gastric**		DM2		Autoinfl.		**TOTAL**	
**Patients**	**Pathology**	**N**	**(%)**	N	(%)	N	(%)	N	(%)	N	(%)	N	(%)	N	(%)	N	(%)	N	(%)	N	(%)	N	(%)	N	(%)	N	(%)	**N**	**(%)**
**Familial**	APS1*	2	28.6	NA	NA	0	0.0	0	0.0	1	50.0	1	50.0	2	100.0	0	0.0	0	0.0	0	0.0	0	0.0	0	0.0	0	0.0	**4**	**2.4**
	APS2*	1	14.3	0	0.0	NA	NA	0	0.0	1	100.0	1	100.0	0	0.0	1	100.0	0	0.0	0	0.0	0	0.0	0	0.0	0	0.0	**3**	**1.8**
	(Graves)	5	71.4	0	0.0	0	0.0	NA	NA	NA	NA	1	20.0	0	0.0	5	100.0	0	0.0	0	0.0	0	0.0	1	20.0	0	0.0	**7**	**4.2**
	(Hashimoto)	23	328.6	1	4.3	1	4.3	NA	NA	NA	NA	3	13.0	5	21.7	13	56.5	8	34.8	3	13.0	5	21.7	4	17.4	5	21.7	**43**	**25.9**
	APS3A	5	71.4	1	20.0	1	20.0	1	20.0	3	60.0	NA	NA	1	20.0	3	60.0	1	20.0	0	0.0	1	20.0	0	0.0	0	0.0	**11**	**6.6**
	(gNETs)	7	100.0	2	28.6	0	0.0	0	0.0	5	71.4	1	14.3	NA	NA	NA	NA	1	14.3	0	0.0	NA	NA	3	42.9	1	14.3	**13**	**7.8**
	(CAG)	19	271.4	0	0.0	1	5.3	5	26.3	13	68.4	3	15.8	NA	NA	NA	NA	4	21.1	2	10.5	NA	NA	3	15.8	1	5.3	**32**	**19.3**
	APS3C	9	128.6	0	0.0	0	0.0	0	0.0	8	88.9	1	11.1	1	11.1	4	44.4	NA	NA	2	22.2	4	44.4	1	11.1	2	22.2	**19**	**11.4**
	APS3D	4	57.1	0	0.0	0	0.0	0	0.0	3	75.0	0	0.0	0	0.0	2	50.0	2	50.0	NA	NA	2	50.0	0	0.0	3	75.0	**10**	**6.0**
	DM II	6	85.7	0	0.0	0	0.0	1	16.7	4	66.7	0	0.0	3	50.0	3	50.0	1	16.7	0	0.0	0	0.0	NA	NA	0	0.0	**12**	**7.2**
	Autoinflamatory	5	71.4	0	0.0	0	0.0	0	0.0	5	100.0	0	0.0	1	20.0	1	20.0	2	40.0	3	60.0	3	60.0	0	0.0	NA	NA	**12**	**7.2**
	non-gastric**	5	*	0	0.0	0	0.0	0	0.0	5	100.0	1	20.0	NA	NA	NA	NA	4	80.0	2	40.0	NA	NA	0	0.0	3	60.0	**15**	*
	**TOTAL**	86		4		3		7		43		11		13		32		19		10		15	*	12		12		166	
**Sporadic**	APS1*	0	0.0	NA	NA	0	0.0	0	0.0	0	0.0	0	0.0	0	0.0	0	0.0	0	0.0	0	0.0	0	0.0	0	0.0	0	0.0	**0**	**0.0**
	APS2*	1	33.3	0	0.0	NA	NA	0	0.0	1	100.0	0	0.0	1	100.0	0	0.0	0	0.0	0	0.0	0	0.0	0	0.0	0	0.0	**2**	**10.0**
	(Graves)	2	66.7	0	0.0	0	0.0	NA	NA	NA	NA	0	0.0	0	0.0	2	100.0	0	0.0	0	0.0	0	0.0	0	0.0	0	0.0	**2**	**10.0**
	(Hashimoto)	4	133.3	0	0.0	1	25.0	NA	NA	NA	NA	1	25.0	2	50.0	2	50.0	0	0.0	0	0.0	0	0.0	0	0.0	0	0.0	**6**	**30.0**
	APS3A	1	33.3	0	0.0	0	0.0	0	0.0	1	100.0	NA	NA	0	0.0	1	100.0	0	0.0	0	0.0	0	0.0	0	0.0	0	0.0	**2**	**10.0**
	(gNETs)	3	100.0	0	0.0	1	33.3	0	0.0	2	66.7	0	0.0	NA	NA	NA	NA	0	0.0	0	0.0	NA	NA	0	0.0	0	0.0	**3**	**15.0**
	(CAG)	4	133.3	0	0.0	0	0.0	2	50.0	2	50.0	1	25.0	NA	NA	NA	NA	0	0.0	0	0.0	NA	NA	0	0.0	0	0.0	**5**	**25.0**
	APS3C	0	0.0	0	0.0	0	0.0	0	0.0	0	0.0	0	0.0	0	0.0	0	0.0	NA	NA	0	0.0	0	0.0	0	0.0	0	0.0	**0**	**0.0**
	APS3D	0	0.0	0	0.0	0	0.0	0	0.0	0	0.0	0	0.0	0	0.0	0	0.0	0	0.0	NA	NA	0	0.0	0	0.0	0	0.0	**0**	**0.0**
	DM II	0	0.0	0	0.0	0	0.0	0	0.0	0	0.0	0	0.0	0	0.0	0	0.0	0	0.0	0	0.0	0	0.0	NA	NA	0	0.0	**0**	**0.0**
	Autoinflamatory	0	0.0	0	0.0	0	0.0	0	0.0	0	0.0	0	0.0	0	0.0	0	0.0	0	0.0	0	0.0	0	0.0	0	0.0	NA	NA	**0**	**0.0**
	non-gastric**	0	*	0	0.0	0	0.0	0	0.0	0	0.0	0	0.0	0	0.0	0	0.0	0	0.0	0	0.0	NA	NA	0	0.0	0	0.0	**0**	*
	**TOTAL**	**15**		**0**		**2**		**2**		**6**		**2**		**3**		**5**		**0**		**0**		**0**	*	**0**		**0**		**20**	

P: Positivity; NA: not applicable

*APS1 and APS2 are presented but not considered for discussion due to the low number of patients

**Associations of non-gastric patients were not considered for the total associations.
