# Supplementary material for: Alterations in SLC4A2, SLC26A7 and SLC26A9 Drive Acid–Base Imbalance in Gastric Neuroendocrine Tumors and Uncover a Novel Mechanism for a Co-Occurring Polyautoimmune Scenario
Source: Cells. 2021 Dec 10;10(12):3500. doi: 10.3390/cells10123500 (PMC8700745; doi:10.3390/cells10123500)
Supplement: Supplementary file 1 [file cells-10-03500-s001.zip › Supplementary Materials.pdf]

Supplementary materials

Samples

Genomic DNA for genetic studies (WES, tNGS and segregation) was isolated from peripheral blood using the FlexiGene DNA Kit (QIAGEN) following the manufacturer's instructions. RT-qPCR for expression studies was performed to test the expression of genes from the panel in the tissues of interest. cDNA was obtained by reverse transcription of 1200 ng of total mRNA using the High Capacity cDNA Reverse Transcription Kit (Applied Biosystems #4368814) following the manufacturer's instructions. RNA from normal tissue of healthy patients was extracted from Formalin-Fixed Paraffin-Embedded tissues using the DNeasy® Blood & Tissue Kit (Qiagen, Cat. No. 69504) following the manufacturer's instructions. PCR reactions were carried out with ~25 ng of cDNA and POWER SYBR® green PCR Master Mix (Applied Biosystems #4367659).

Discovery series WES1

Exomes were captured and enriched using the SureSelect Human All Exon Kit (78 Mb) (Agilent Technologies, Santa Clara, CA). Enriched samples were paired-end sequenced on an Illumina Genome Analyzer II sequencing platform using 2 lanes per sample and generating 101 base-pair long reads. FAST files of short reads were aligned to the GRCh38/hg19 reference genome using the BWA algorithm. GATK-based variant calling was performed for aligned reads considering DP (Read Depth) values of >30 and Quality-by-Depth scores for a variant confidence of >1.00. Strict filtering for only well-defined variants by quality controls, and those not included in repeat regions were included to prevent false positives. Variants with low coverage (<6x) and/or low frequency of the alternative variant allele (<10%) were discarded. Only quality-filtered variants affecting coding sequences of canonical transcripts (non-synonymous, essential splice site, frame shift or gain/loss of stop codons) were taken into account. Variant type annotation, population statistics, disease-specific sequence databases, and in silico prediction algorithms were applied according to AMCG standards and guidelines [1]. Variants with a minor allele frequency (MAF) <0.03 (dbSNP130, 1000 Genomes and ExAC) were considered. Their potential damaging effect was assessed using the VEP [2] script software package (including Sift, Polyphen and Condel damage predictors). Only putatively damaging variants by in silico assessment were selected.

Genes with filtered variants from patients were investigated together in an enrichment study to uncover candidate genes involved in the thyrogastric syndrome. Two different software packages were used for independent assessments of the gene set analyses. Data were analyzed with Qiagen's Ingenuity Pathway Analysis (IPA, Qiagen, Redwood City, CA, www.qiagen.com/ingenuity) and

ConsensusPathDB (available at http://cpdb.molgen.mpg.de/) [3]. High-confidence induced network module analysis without intermediate nodes were evaluated considering biochemical, regulation and genetic interactions. Previously described achlorhydria genes (*ATP4A* and *PTH1R)* were included in the analysis with the new genes to highlight achlorhydria regulation process.

Whole exome sequencing data have been deposited in RD-Connect GPAP platform from the CNAG (National Center for Genomic Analysis) available at https://platform.rd-connect.eu/genomics/.

In vitro Studies

DNA constructs and cloning of guide RNAs for SLC26A7, SLC26A9 and SLC4A2 genes:

The online Benchling CRISPR gRNA Design tool (http://www.benchling.com) was used for the selection of two sgRNA with high specificity rank and low potential off-target score for *SLC26A7, SLC26A9* and *SLC4A2* target genes. Individual sgRNAs were cloned into a modified LentiCRISPRv2 lentiviral expression vector (Addgene #52961) using annealed oligonucleotides containing overhanging ends compatible BsmBI sites (NEB) as previously described (PMID: 25075903). LentiCRISPRv2 was modified to express different selection antibiotics (Blasticidin and Hygromycin) for the combination of different sgRNAs in the same cell line. The sgRNAs and oligo sequences are listed in Supplemental Table S5.

Lentivirus generation, titration, transduction and antibiotic selection:

Recombinant lentiviruses were produced by transient plasmid transfection of HEK293T/17 cells (PMID: 28494941). Briefly, one day before transfection cells were seeded at 12×106 cells/dish in 15-cm dishes; next day, cells were transfected by the calcium-phosphate method using 14.6 and 7.9 ug of second-generation lentiviral packaging plasmids (psPAX2 and pMD.2G, Addgene #12260 and #12259, respectively) and 22.5 ug of the CRISPR transfer plasmid. The supernatant was collected 48 hours later, cleared by low-speed centrifugation (1500rpm at room temperature), and filtered using low-protein retention filters (0.45-um PVDF filters, Millipore). Viral titers were calculated by qRT-PCR analysis on transduced HEK293T cells. Viral aliquots were stored at -80 °C.

HEK293T cells were re-plated 24 h before transduction and transduced using a multiplicity of infection (MOI) of 5. After transduction, cells were maintained for 48 hours in normal media and after this period the proper antibiotic was added at the previously tested concentration, Hygromycin 100 ug/mL, Puromycin 1ug/mL and Blasticidin 5ug/mL. Cells were selected for 1 week and change back to normal media after this period. Genomic analysis was carried out to confirm proper genomic

targeting. Disruption of the gene was confirmed by Sanger sequencing. Primers are listed in Supplemental Table S5. DNA from each cell line was extracted using the DNeasy Blood and Tissue (Qiagen).

Colony-forming assay:

100x cells were seeded in 6-well plates in triplicate. 10 days after plating, colonies were fixed in methanol and stained with 0.5 M of crystal violet (Alfa Aesar, Cat. B21932). Number and diameter of colonies per cell line was measured by using Fiji software (available under GNU General Public License at https://fiji.sc/).

Flow cytometry:

For all the experiments, cells were seeded onto 100mm plates with enriched and restrictive medium for 24 hours. For cell cycle experiments, cells were fixed in 100% ethanol for 24 houres and then washed 2x by phosphate-buffered saline (PBS); RNAse (Quiagen) and treated by adding Propidium Iodide. For TMRE (tetramethylrhodamine ethyl ester) studies, cells were trypsinized with the supernatant, washed with PBS, and incubated with 40 nM TMRE (Sigma) for 10 minutes at 37ºC; they were after washed in PBS, and DAPI was added. For DCFDCA studies, cells were tripsinized and resuspended in pre-warmed 5 uM DCFDA (CM-H2DCFDA, Thermofisher) for 30 minutes and washed in PBS. Samples were acquired on a FACS Canto II (Beckton Dickinson). Cell cycle phases separation was performed by using Hoechst33342 and Pyronin. Hoechst is an exclusive DNA dye, while Pyronin Y reacts with both DNA and RNA. In the presence of Hoechst, Pyronin Y reaction with DNA is blocked, and Pyronin Y stains RNA only. Quiescent cells (G0 phase), have lower level of RNA compared to active cells (G1 phase). Cells were fixed with 70% ethanol, washed with HBSS and resuspended in HBSS containing 1 ug/ml Hoechst33342 and incubated at 37ºC for 30 minutes. Pyronin (Sigma Aldrich) was added at a final concentration of 0.5uM and incubated at 37ºC for 15 minutes. All data and gating were analyzed using FlowJo 9.9.4 (Treestar, Oregon).

Targeted Next Generation Sequencing (tNGS)

Twenty-five ng of index case genomic DNA was tagmented and amplified. Quality and quantity of purified libraries were assessed with an Agilent 2100 Bioanalyzer. Libraries were sequenced on a MiSeq platform from Illumina. Reads were aligned to the human reference genome version GRCh38/hg38 using BWA aligner. Low-quality reads and PCR duplicates were removed from the BAM-formatted file. Variant calling was performed using a combination of VarScan and GATK, along with in-house scripts to combine and filter variants. Only variants in coding regions

with a MAF <0.03 were considered. Only putatively damaging variants by in silico assessment were selected.

The genomes of 69 patients affected with co-occurring immunodeficiencies were sequenced by tNGS using the custom panel. In addition, 40 healthy Spanish individuals were included in the tNGS study. Variants found in both patients and controls were discarded. The candidate variants were confirmed by Sanger sequencing in the proband and family members in segregation studies.

Efficacy (Positivity) studies were performed including the results of 76 patients belonging to the series of 69 APS patients from the tNGS studies, the 5 families from the Discovery WES1, and the F1 and F2 families previously published [4][5]. Efficacy was calculated per allele in order to differentiate heterozygous for one or two variants and homozygous states. A homozygous variant or two variants in two different genes was considered as two positive alleles (P+), while a single heterozygous variant in one gene was considered as partially positive (P+/-). Patients were considered panel-negative (P-) when no variants were found. Thus, positivity counted two positive alleles for P(+) patients and one positive allele for P(+/-) patients.

Supplementary references

1. Richards, S., Aziz, N., Bale, S., Bick, D., Das, S., Gastier-Foster, J., et al. Standards and guidelines for the interpretation of sequence variants: A joint consensus recommendation of the American College of Medical Genetics and Genomics and the Association for Molecular Pathology. Genetics in Medicine. 2015;17:405–24.
2. McLaren, W., Pritchard, B., Rios, D., Chen, Y., Flicek, P., Cunningham, F. Deriving the consequences of genomic variants with the Ensembl API and SNP Effect Predictor. Bioinformatics. 2010;
3. Kamburov, A., Stelzl, U., Lehrach, H., Herwig, R. The ConsensusPathDB interaction database: 2013 Update. Nucleic Acids Research. 2013;41.
4. Calvete, O.; Reyes, J.; Zuñiga, S.; Paumard-Hernández, B.; Fernández, V.; Bujanda, L.; Rodriguez-Pinilla, M.S.; Palacios, J.; Heine-Suñer, D.; Banka, S.; et al. Exome sequencing identifies ATP4A gene as responsible of an atypical familial type I gastricneuroendocrine tumour. *Hum. Mol. Genet.* **2015**, doi:10.1093/hmg/ddv054.
5. Calvete, O.; Herraiz, M.; Reyes, J.; Patiño, A.; Benitez, J. A cumulative effect involving malfunction of the PTH1R and ATP4A genes explains a familial gastric neuroendocrine tumor with hypothyroidism and arthritis. *Gastric Cancer* **2017**, doi:10.1007/s10120-017-0723-8.
